# A biosensor for the direct visualization of auxin

**DOI:** 10.1038/s41586-021-03425-2

**Published:** 2021-04-07

**Authors:** Ole Herud-Sikimić, Andre C. Stiel, Martina Kolb, Sooruban Shanmugaratnam, Kenneth W. Berendzen, Christian Feldhaus, Birte Höcker, Gerd Jürgens

**Affiliations:** 1 Max Planck Institute for Developmental Biology, Tübingen, Germany; 2 Institute for Biological and Medical Imaging, Helmholtz Zentrum Munich, German Research Center for Environmental Health, Munich, Germany; 3 Department of Biochemistry, University of Bayreuth, Bayreuth, Germany; 4 Centre for Plant Molecular Biology, University of Tübingen, Tübingen, Germany

**Keywords:** Optical imaging, Cellular imaging, Auxin, Plant physiology

## Abstract

One of the most important regulatory small molecules in plants is indole-3-acetic acid, also known as auxin. Its dynamic redistribution has an essential role in almost every aspect of plant life, ranging from cell shape and division to organogenesis and responses to light and gravity^1,2^. So far, it has not been possible to directly determine the spatial and temporal distribution of auxin at a cellular resolution. Instead it is inferred from the visualization of irreversible processes that involve the endogenous auxin-response machinery^3–7^; however, such a system cannot detect transient changes. Here we report a genetically encoded biosensor for the quantitative in vivo visualization of auxin distribution. The sensor is based on the *Escherichia coli* tryptophan repressor^8^, the binding pocket of which is engineered to be specific to auxin. Coupling of the auxin-binding moiety with selected fluorescent proteins enables the use of a fluorescence resonance energy transfer signal as a readout. Unlike previous systems, this sensor enables direct monitoring of the rapid uptake and clearance of auxin by individual cells and within cell compartments in planta. By responding to the graded spatial distribution along the root axis and its perturbation by transport inhibitors—as well as the rapid and reversible redistribution of endogenous auxin in response to changes in gravity vectors—our sensor enables real-time monitoring of auxin concentrations at a (sub)cellular resolution and their spatial and temporal changes during the lifespan of a plant.

## Abstract (web summary)

A genetically encoded sensor for the quantitative visualization of auxin distribution in plants enables real-time monitoring of its uptake and clearance by individual cells and within cellular compartments.

## Main

The tryptophan-derived metabolite indole-3-acetic acid (IAA) has an important role in plants, triggering a multitude of developmental processes and responses to environmental cues^1,2^. Much progress has been made towards a mechanistic understanding of the nuclear events that transform auxin perception into transcriptional responses^9–11^. Other studies have investigated the basic machinery involved in the polar and non-vectorial release of auxin from the cell—which occurs through the action of PINFORMED efflux transporters and ABCB transporters— within a tissue context, and have resulted in computer models of how canalized auxin flow mediates developmental or physiological processes^12–14^. By contrast, owing to technical limitations (reviewed in ref. ^15^), very little is known about the actual distribution of auxin in tissues at single-cell resolution. At present, plant biologists can use only proxies to visualize auxin distribution, such as the auxin-dependent expression of reporter genes (for example, using the systems DR5::GUS^3^; DR5::ER-GFP^4^ and DR5::NLS-3xGFP^5^). However, this indirect approach is characterized by latencies and can be affected by modulation of the auxin signalling machinery. More recently, IAA levels have been inferred from auxin-dependent degradation—and thus signal reduction—of fluorescent proteins linked to domain II of an IAA inhibitory protein (for example, DII-VENUS^6^ and R2D2^7^). A limitation of these approaches is their irreversibility, which precludes the visualization of transient changes in auxin levels.

The ideal sensor for the visualization of auxin dynamics in planta should have the following features: first, physical interaction of the sensor with auxin should elicit a fluorescent signal in a reversible manner, so that changes in auxin concentration can be monitored; second, the sensitivity of the sensor should be sufficiently high to image the dynamic auxin distribution over time; third, the sensor should be targetable to different subcellular compartments—locations that are out of reach for the conventional proxies, which rely on gene expression or protein degradation; and fourth, the sensor should not contain components that are involved in plant metabolism or regulation, such that both interference with auxin responses and regulation of the sensor by the plant are avoided.

**Fig. 1:**
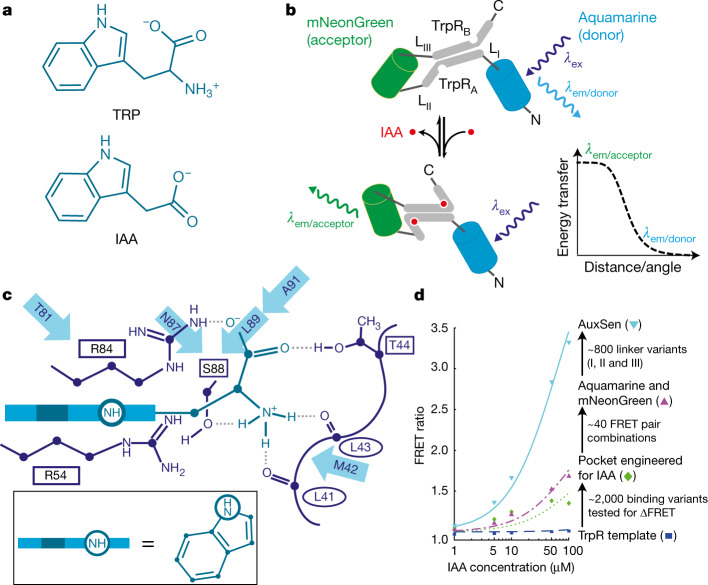
Summary of the design process. **a**, Chemical structures of TRP and IAA. **b**, Principle of the sensor design. Only in the presence of IAA (red) are the fluorophores (mNeonGreen and Aquamarine) sufficiently close and in the correct orientation for energy transfer (*E*_FRET_). N and C represent the N and C termini of the proteins, respectively; L represents the linker; and *λ*_ex_ and *λ*_em_ represent the excitation and emission wavelengths, respectively. **c**, Structure of the binding pocket of TrpR with ligand in side view (boxed) (modified from ref. ^8^). Interactions with the side chains of R84, S88 and T44 (second TrpR chain) as well as the backbone carbonyl groups of L41 and L43 (second TrpR chain) are shown explicitly. Further residues that were mutated in this study are indicated with arrows. **d**, Major steps in the design of the sensor (AuxSen), and their cumulative contribution to the change in FRET ratio (ΔFRET) plotted against IAA concentration . Template sensor construct, TrpR–eCFP–Venus (blue squares); engineered binding pocket for IAA, TrpR(M42F/T44L/T81M/N87G/S88Y)–eCFP–Venus (green diamonds); optimized fluorophore combination, TrpR(M42F/T44L/T81M/N87G/S88Y)–mNeonGreen–Aquamarine (purple triangles); AuxSen, TrpR(M42F/T44L/T81M/N87G/S88Y)–mNeonGreen–Aquamarine with optimised linkers I, II and III (light blue inverted triangles).

**Extended Data Fig. 1:**
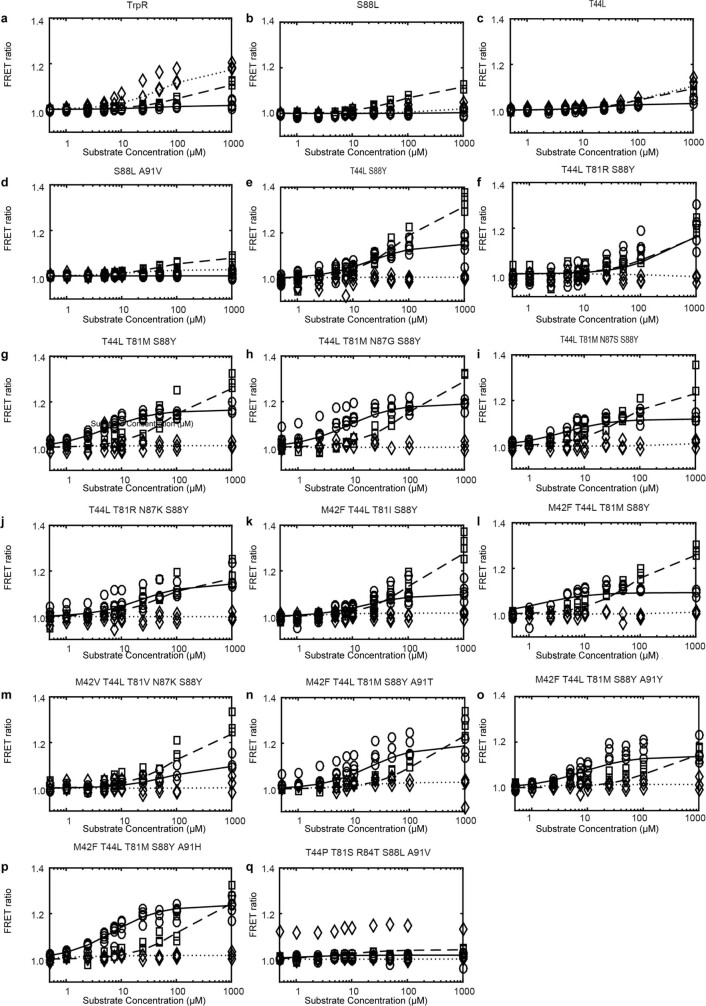
FRET response of several binding-domain variants to increasing substrate concentration. **a**, Wild-type TrpR. **b**–**q**, Engineered TrpR variants. Each mark indicates a single measurement. Circle, IAA; rhombus, TRP; square, IAN.

With these boundary conditions in mind, we developed a genetically encoded, fully reversible biosensor for in vivo imaging of auxin gradients with high spatial and temporal resolution, starting from the bacterial tryptophan repressor (TrpR). IAA resembles tryptophan: both contain an indole ring, the 3-position of which is connected to an amino acid moiety in TRP and a carboxyl group in IAA (Fig. 1a). The dimeric TrpR undergoes a conformational change upon binding TRP^16,17^, and fluorescent proteins fused to TrpR can relay this change, generating a fluorescence resonance energy transfer (FRET) signal as a convenient readout for in vivo measurements^18^ (Fig. 1b). Furthermore, TrpR exhibits low affinity towards IAA^8^. This makes TrpR an ideal starting point for developing an auxin-specific, genetically encoded FRET biosensor^19^. Our design efforts were aimed at improving the affinity and specificity of IAA binding, while abolishing TRP binding. We assumed a comparable binding mode for the indole ring of both TRP and IAA, and focused our design around the TrpR residues in the vicinity of the amino group of TRP (Fig. 1c), aiming to improve the specificity for the carboxyl group of IAA. This selection was later expanded to include adjacent residues. Altogether, 2,000 variants were generated in successive rounds of mutagenesis and were screened for an increase in FRET signal upon the addition of IAA (Fig. 1d, Extended Data Fig. 1). Improved variants were checked for ligand specificity using a library of substances that are similar to IAA and are reportedly present in *Arabidopsis* (Extended Data Table 1). To confirm improvements in binding affinity, selected TrpR variants were analysed by isothermal titration calorimetry (Extended Data Table 2a). Furthermore, the structures of several variants were elucidated by X-ray crystallography, to guide mutagenesis experiments (Extended Data Table 2b, Supplementary Table 1a–h).

**Fig. 2:**
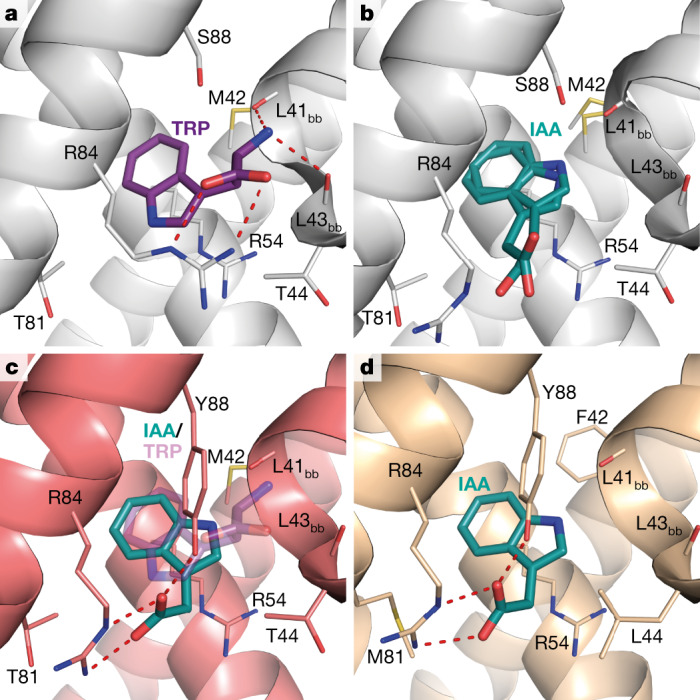
Structure of AuxSen and critical steps in the engineering process. **a**, **b**, Structure of TrpR bound to the native ligand TRP (purple, Protein Data Bank (PDB) ID: 1ZT9) (**a**) and to the design-target IAA (green) (**b**). IAA is rotated by 180° in the binding pocket compared with TRP. Owing to a lack of stabilization when binding to TrpR, IAA shows conformational freedom; two alternative conformations are shown. **c**, The mutation S88Y sterically precludes the positioning of TRP (transparent purple) in the binding site while favouring the binding of IAA. **d**, Structure of the final AuxSen variant (TrpR(M42F/T44L/T81M/N87G/S88Y)) bound to IAA. The ligand is firmly packed in the enhanced hydrophobic pocket of TrpR and is anchored to R84 as well as Y88, resulting in a high affinity of AuxSen for IAA. All structures are superimposed on the Cα of residues 20–60 of both chains. Red dashed lines show polar interactions between ligand and side-chain atoms. The subscript ‘bb’ labels residues that have interactions of backbone atoms with the ligand.

**Extended Data Fig. 2:**
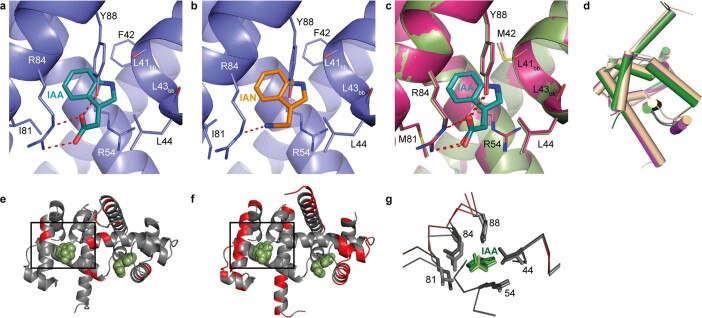
Details observed in the crystal structures. **a**, Structure of IAA in the binding pocket of TrpR(M42F/T44L/T81I/S88Y). **b**, Structure of IAN bound to the same variant as in **a**. **c**, Overlay of IAA in TrpR(T44L/T81M/S88Y) (magenta) and TrpR(T44L/T81M/N87G/S88Y) (green). **d**, Structural overview of variants TrpR(T44L/T81M/N87G/S88Y) (green), TrpR(T44L/T81M/S88Y) (magenta), and TrpR(M42F/T44L/T81M/N87G/S88Y) (AuxSen, gold). It is apparent that AuxSen differs from the two intermediate structures regarding the overall arrangement of the helices. **e**–**g**, The structure of TrpR(S88Y/T44L) and all variant structures based on it show a slight relocation of the backbone of residues 70–90. This is probably due to the fact that all structures based on this variant crystallize in the orthorhombic space group *P*2_1_2_1_2_1_ (**f**) as opposed to the tetragonal space group *P*4_3_ found for TrpR–IAA and TrpR(S88Y)–IAA (**e**). Both geometries have been found in earlier crystal structures of TrpR, for example PDB 1ZT9 (tetragonal) and 2OZ9 (orthorhombic). It seems that the introduction of the T44L mutation strongly favours crystallization in the orthorhombic geometry. In the *P*2_1_2_1_2_1_ space group, crystals form more extensive crystal contacts. The structure overlay (**g**) shows how several residues are displaced (residues that have symmetry mates within 3 Å are shown in red, and the ligand IAA is shown in green). However, interactions and positioning of the ligands are maintained. Nonetheless, we only compare backbone coordinates between variants of the same space group, to exclude misinterpretations due to crystal contacts.

**Extended Data Fig. 3:**
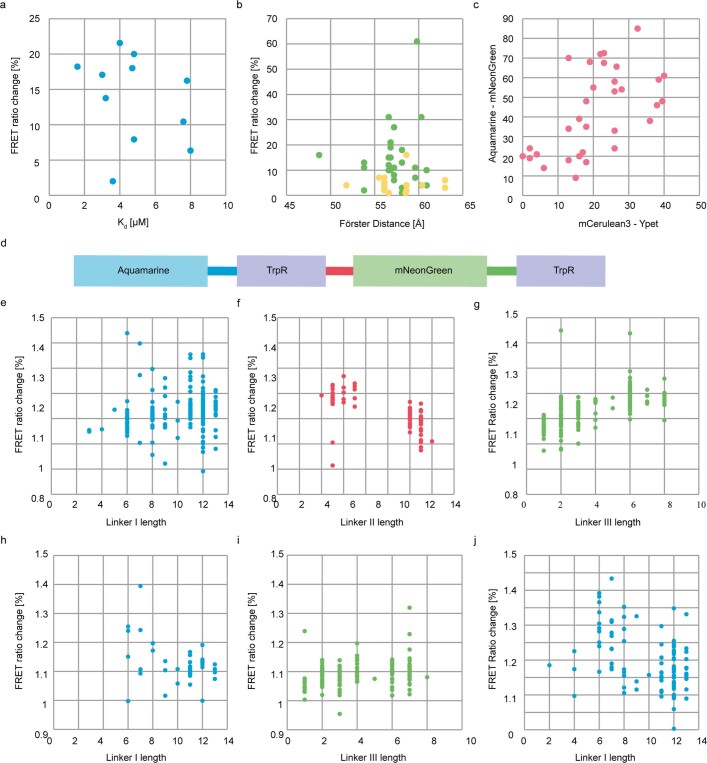
Parameters tested for potential influence on the change of FRET ratio. **a**, Change in FRET ratio upon IAA treatment plotted against the dissociation constant (*K*_d_) of the same variant as determined by ITC. **b**, FRET ratio changes do not correlate with the Förster distance. Blue–yellow pairs are marked in green, yellow–red pairs are in orange. Blue–yellow pairs, in general, show a higher FRET ratio change upon IAA treatment, but a similar range of Förster distances as the yellow–red ones. **c**, FRET ratio changes (in per cent) of several variants tested with two different fluorophore pairs. Variants showing a strong response with one fluorophore pair usually also show a strong response with another pair (correlation coefficient = 0.6). **d**–**j**, Effects of mutations in linkers. **d**, Structure of the construct. The IAA-binding TrpR variants were cloned as tandem repeats into the construct containing donor and acceptor fluorophores, analogous to ref. ^18^. The positional effect of the fluorescent proteins probably stems from slight rearrangements of the overall backbone in both TrpR subunits. Predominantly, this involves helix E of the reading-head motif, which mediates the DNA interaction in the natural function of TrpR. Because helix E is towards the C-terminal end of the chain, is it thought that fluorescent proteins positioned at this end will experience a larger positional relocation and thus show a more dynamic range of the FRET signal. **e**–**g**, First-round linker mutations. All three linkers were mutated, but no pattern for the optimal linker length could be determined. One linker II variant was chosen for further mutations. **h**, **i**, Second-round linker mutations. Linkers I and III were mutated in the variant obtained in the first round, with no changes in the optimized linker II. **j**, Third-round linker mutations. Linker I was further mutated in the variant containing mutations in linkers II and III. The linker length axis indicates the number of amino acid residues.

Our structural analysis showed that, when binding to TrpR, IAA is flipped by 180° compared with TRP (Fig. 2a), with the carboxyl group of IAA facing the opening of the TrpR binding pocket (Fig. 2b). TRP is anchored by interactions with the surrounding protein residues, whereas IAA binding shows no such stabilization, which is reflected in the poor binding affinity of this interaction (Extended Data Table 2a). In engineering the auxin sensor, we identified variants that stabilize and favour this IAA-binding mode. Primarily, a serine-to-tyrosine mutation at position 88 (S88Y) was found to entirely block TRP binding owing to the bulky side chain of Y88, while simultaneously favouring IAA binding through interaction of the carboxyl group of IAA with the guanidino group of R84 and the hydroxyl group of Y88 (Fig. 2c). The affinity for IAA was improved further by optimizing hydrophobic interactions of its indole ring with the TrpR binding pocket; to this end, the mutations T44L and T81M were incorporated in the final sensor design (Fig. 2d). During the engineering process, we also monitored the binding of indole-3 acetonitrile (IAN)—which could potentially compete with IAA—to TrpR (Extended Data Table 2a). The binding modes of IAN and IAA are markedly similar (Extended Data Fig. 2a, b); however, mutations such as N87G exert discriminating effects through small changes in the positioning of Y88 (Extended Data Fig. 2c). Finally, we identified mutations that have no favourable effect on IAA affinity but improve the FRET readout—probably through changes in the packing, and therefore the orientation, of the attached fluorescent proteins (Extended Data Fig. 2d). We then optimized the fluorophores and the linker combinations (Extended Data Fig. 3) to yield our final sensor, which we term ‘AuxSen’, with the composition mNeonGreen–TrpR–Aquamarine–TrpR, in which TrpR is TrpR(M42F/T44L/T81M/N87G/S88Y) (Figs. 1d, 2d).

**Extended Data Fig. 4:**
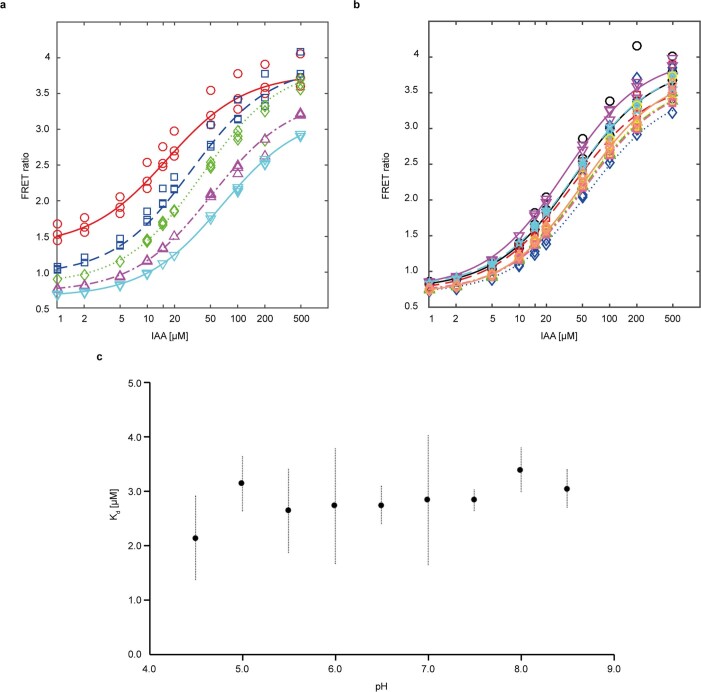
pH, salt and redox sensitivity of AuxSen. **a**, The FRET ratio is slightly affected by changes in the pH, but fully functional in the range of pH values within the plant cell. Red, pH 6.0; blue, pH 6.5; green, pH 7.0; violet, pH 7.5; cyan, pH 8.0. **b**, The FRET ratio is not strongly affected by salts and changes in the redox potential. Black, control; red, 1 mM (NH_4_)_2_SO_4_; blue, 1 mM CaCl_2_; green, 10 mM NH_4_NO_3_; violet, 10 mM DTT; cyan, 10 mM H_2_O_2_; yellow, 10 mM KCl; orange squares, 10 mM KNO_3_; orange diamonds, 10 mM NaCl. **c**, ITC data of AuxSen binding to IAA, measured at different pH values. Data are mean ± s.d., derived from 3 technical replicates.

**Extended Data Fig. 5:**
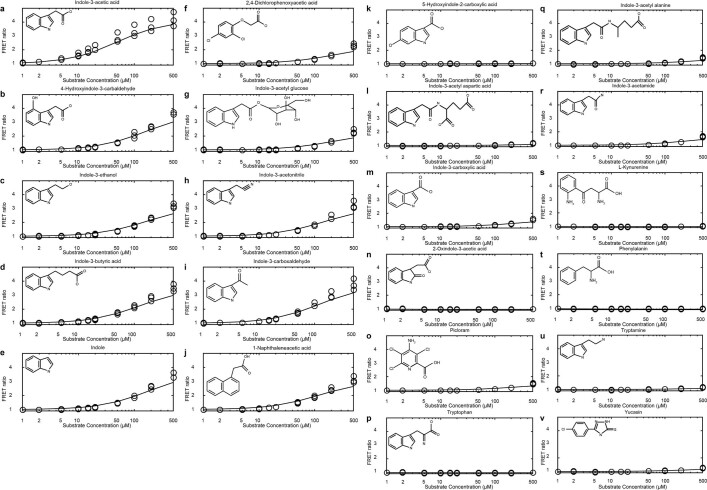
Affinities of AuxSen for auxin-related compounds. **a**–**j**, Compounds with weak affinities; **k**–**v**, compounds with no affinity. The change in FRET ratio (*y* axis) is plotted against increasing concentrations of the individual compounds in μM (*x* axis). Each mark indicates a single measurement.

In vitro, the FRET ratio of AuxSen changed by a factor of three upon treatment with 50 μM IAA, which is within the range of cellular auxin concentrations^20^. The signal was stable at cytosolic pH, and in the presence of reducing or oxidizing environments and all tested salt ions (Extended Data Fig. 4). The specificity of AuxSen for IAA was assessed using other indole derivatives. Among these, AuxSen showed the highest affinity for IAA; although a response was observed for other compounds, their binding affinities were reduced by around one order of magnitude (Extended Data Fig. 5). Of these indole derivatives, only IAN is present in substantial amount in plants (Extended Data Table 1). However, roots show a growth response to treatment with IAN, and modelling suggests that the IAA receptor SCF^TIR1^ could bind IAN^21^. Thus, IAN is probably sequestered and is therefore unlikely to interfere with auxin sensing in the plant.

**Fig. 3:**
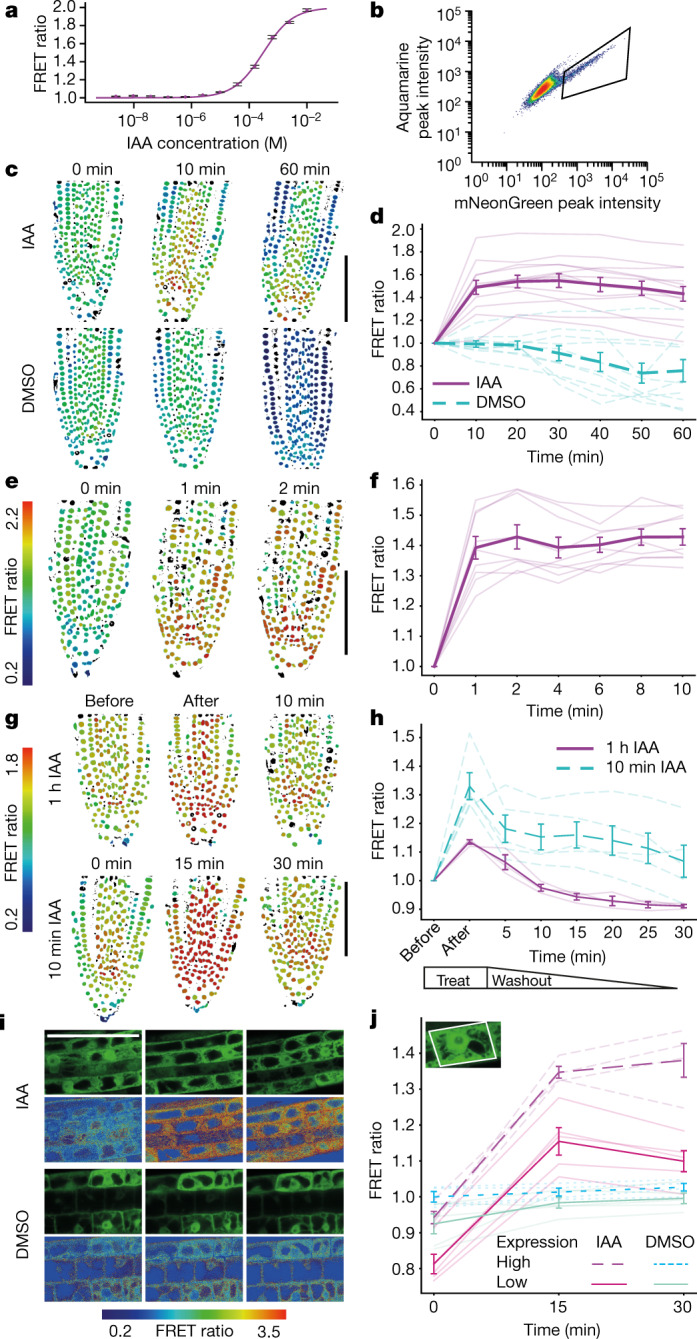
FRET ratio of AuxSen in response to auxin treatment. **a**, **b**, The FRET ratio obtained by flow cytometry in *Arabidopsis* protoplasts. **a**, Dose–response curve, normalized to the minimum FRET ratio (mean ± s.e.m.; *n* = 3 biologically independent samples). **b**, Baseline fluorescence intensity (‘Log_Height’ (a.u.)) without exogenous IAA; the relevant area is boxed (Extended Data Figs. 6,7). **c**–**f**, Changes in the FRET ratio (colour bar) in root nuclei incubated in 10 μM IAA recorded for 1 h (**c**, **d**) or for 10 min (**e**, **f**). **c**, **e**, Images (DMSO, control). Scale bar, 100 μm. **d**, **f**, Quantitative analyses. The thick lines represent the mean (±s.e.m.), and the thin lines each represent independent single-seedling measurements (*n* = 14 (experimental), *n* = 10 (control) in **d**, *n* = 9 in **f**). **g**, **h**, Changes in the FRET ratio (colour bar) in root nuclei following the washout of IAA. **g**, Images obtained after incubation with 10 μM IAA for 1 h (top) or 10 min (bottom). Images were taken before or immediately after IAA treatment, or 10 min after the end of IAA treatment. Scale bar, 100 μm. **h**, Quantitative analysis. The thick lines represent the mean (±s.e.m.), and the thin lines each represent independent single-seedling measurements (*n* = 5 (10 min), *n* = 3 (1 h)). **i**, **j**, Change in the FRET ratio of ER-localized AuxSen in response to 100 μM IAA. **i**, Expression of ER-localized AuxSen (green; first and third rows) and FRET ratio (colour bar; second and fourth rows) of root tissue treated with IAA (top two rows) or DMSO (bottom two rows; control). Scale bar, 50 μm. **j**, Quantitative analysis of cells with high (broken lines; IAA, *n* = 4; DMSO, *n* = 4) or low (solid lines; IAA, *n* = 5; DMSO, *n* = 5) levels of AuxSen accumulation. The thick lines represent the mean (±s.d.), the thin lines each represent individual cells. Inset, AuxSen expression in the ER.

**Extended Data Fig. 6:**
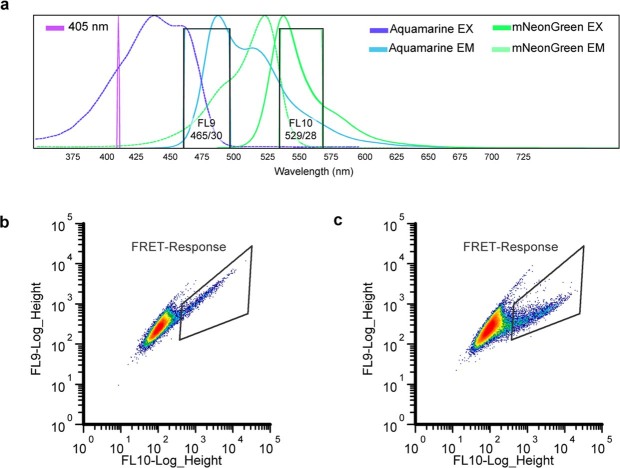
FRET ratio of AuxSen quantified in protoplasts. Cell-suspension-culture protoplasts were transfected with *pJIT60-2xp35SS:NLS:AuxSen* and the FRET response was captured by flow cytometry as the ratio of the emission between the Aquamarine peak intensity (FL9) and mNeonGreen peak intensity (FL10) as ‘Log_Height’ (a.u.). **a**, Normalized excitation and emission spectra for mNeonGreen and Aquamarine. The 405-nm laser line is shown as a vertical bar and bandpass filters are shown as open boxes, as rendered by FPBase^44^. As FRET occurs, the emission output is shifted from the FL9 to the FL10 bandpass. **b**, Baseline level of AuxSen FRET response, without exogenous IAA. **c**, Maximal response level of AuxSen with 10 mM IAA.

**Extended Data Fig. 7:**
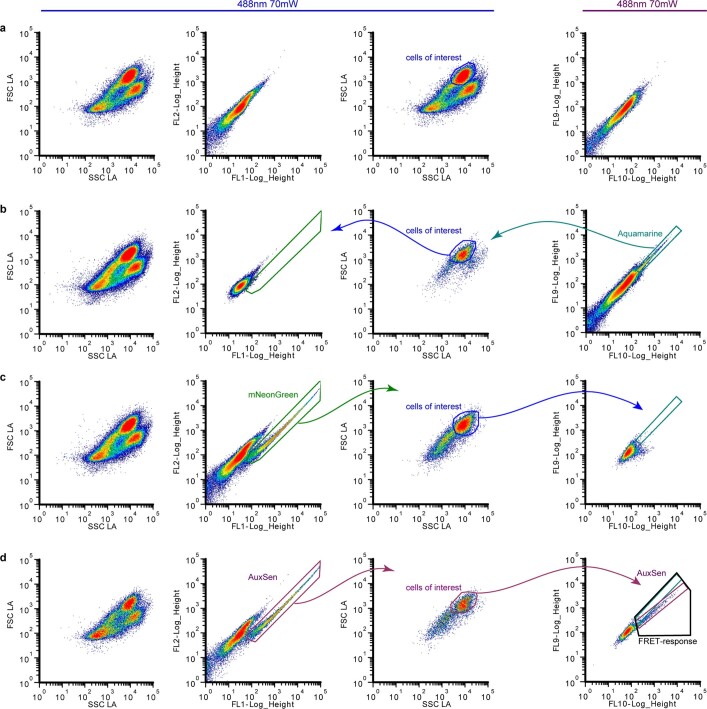
Demonstration of relevant population regions and Aquamarine, mNeonGreen and AuxSen fluorescent emission in cell-suspension-culture protoplasts. Bivariate plots from left to right are as follows: forward versus side-scatter log area ungated; emission peak to shoulder FL1 (534/30) versus FL2 (585/29); forward versus side-scatter log area with cells of interest marked and gated for **b**–**d**; emission peak-to-shoulder FL9 (465/30) versus FL10 (529/28). Arrows indicate ‘gating’, meaning that the following plot is restricted to those data points that fall within that particular window. **a**, Cells only transfected with water. **b**, Cells transfected with *pJIT60-2xp35SS:NLS:Aquamarine*. Cells expressing Aquamarine were used to determine which scattering population produced the fluorescent protein. This gate is followed for mNeonGreen emission. **c**, Cells transfected with *pJIT60-2xp35SS:NLS:mNeonGreen*. Cells expressing mNeonGreen were used to determine which scattering population produced the fluorescent protein. This gate is followed for Aquamarine emission. **d**, Cells transfected with *pJIT60-2xp35SS:NLS:AuxSen*. Cells expressing AuxSen identified by their mNeonGreen emission then restricted to the ‘cells of interest’ scattering population that produced the greatest amount of protein. The FRET-response region was then made to encompass the entire range of possible fluorescence, including the shift in the FL9 to the FL10 bandpasses. See also Supplementary Table 2. Flow cytometry basic gate statistics and FRET-ratios based on (‘FL10-Log_Height’/‘FL9-Log_Height’) ratio are plotted against time for the final gate FRET response.

As a first step to confirm the functionality of AuxSen in vivo, we expressed a nuclear-localized version of the sensor transiently from the viral *35S* promoter in cell-suspension protoplasts^22^, and quantified the FRET response by flow cytometry. The FRET ratio increased with the auxin concentration in the medium over four orders of magnitude, starting at 3 μM IAA (Fig. 3a, b, Extended Data Figs. 6, 7), with the baseline FRET signal thought to reflect the endogenous level of auxin in the protoplast population. The sensitivity of the sensor could therefore be sufficient to report endogenous auxin levels.

**Extended Data Fig. 8:**
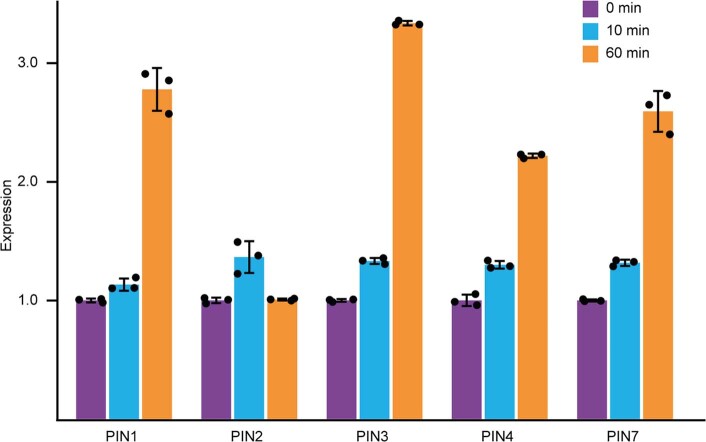
Quantitative analysis of *PIN* mRNA accumulation in seedlings treated with 10 μM IAA. Seedlings were kept in MS medium (control; 0 min, purple) or transferred to a solution containing 10 μM IAA. RNA was extracted from 6 seedlings each after 10 min (blue) or 60 min (orange) and subjected to analysis by quantitative PCR with reverse transcription. Data are mean ± s.d. derived from 3 technical replicates, and black dots represent individual values.

We generated approximately 250 transgenic *Arabidopsis* lines bearing a dexamethasone-inducible expression system (*pBay-bar-pRPS5a-mGAL4-VP16-GR_UAS_NLS_AuxSen*) integrated as a single transgene, and selected 10 lines expressing the sensor stably in the fourth generation. The strong, ubiquitously active promoter *RPS5A* drives expression of the yeast transcription factor Gal4p, the nuclear uptake of which is induced by dexamethasone, and the binding of Gal4p to the *UAS* promoter results in the expression of the nuclear-localized auxin sensor. Strongly expressing lines were identified by mNeonGreen fluorescence in the root tips after dexamethasone induction overnight. To examine the response of AuxSen to auxin in planta, we treated seedlings with 10 μM IAA and recorded the FRET signal over time (Fig. 3c–f). After 10 min, the FRET signal in root nuclei had reached a maximum, and then remained constant for another 50 min; by contrast, treatment with the solvent DMSO did not induce any response (Fig. 3c, d). To reveal the speed of nuclear auxin accumulation, we measured the FRET signal over shorter time intervals. The maximum signal was reached within 2 min, with more than 80% achieved after only 1 min (Fig. 3e, f). The uptake of auxin from the extracellular space therefore seems to be highly efficient. We then investigated the reversibility of nuclear accumulation by washing out IAA. After 10 min of incubation of seedlings in 10 μM IAA, which gave the maximum FRET ratio, the medium was changed to DMSO (Fig. 3g, h). The FRET ratio gradually decreased, almost reaching the pre-incubation value 30 min later, which suggests slow IAA efflux from the cells (Fig. 3g, h). We repeated the experiment but extended the IAA incubation time to 1 h. This accelerated IAA efflux such that the FRET ratio decreased to its pre-incubation level within 10 min of IAA withdrawal (Fig. 3g, h). This increased efflux of IAA correlated with two- to threefold higher expression levels of PIN efflux carriers after 1 h compared to 10 min of IAA incubation (Extended Data Fig. 8). In conclusion, this FRET-based sensor can report the dynamics of transient auxin accumulation. Although traditional reporter systems can detect the response to auxin uptake^6^, the irreversibility of reporter translation or degradation obscures the transient nature of the auxin response. Our data suggest that IAA uptake is a constitutive, fast process, whereas the efflux from the cell occurs on demand, as if auxin regulates its own export.

To explore whether auxin might accumulate in other subcellular compartments—those that cannot be accessed by reporters that are based on gene expression or protein degradation—we targeted AuxSen to the lumen of the endoplasmic reticulum (ER). The localization of PIN and PIN-LIKE putative auxin transporters in the ER membrane has led to speculation that auxin accumulation in the ER could be an ancient mechanism of auxin homeostasis^23^. Incubating seedlings in 100 μM IAA for 15 min led to a strong increase in the FRET ratio compared with the DMSO control, suggesting that IAA is transported into the ER (Fig. 3i, j). Different cells within the same seedling root displayed different levels of AuxSen accumulation in the ER (Fig. 3i). Nonetheless, the change in FRET ratio upon exposure to IAA did not differ substantially between cells expressing higher or lower levels of AuxSen (Fig. 3j). In conclusion, this FRET-based auxin sensor can faithfully report auxin concentrations from a subcellular compartment such as the ER, which is inaccessible to traditional auxin-response reporters.

**Fig. 4:**
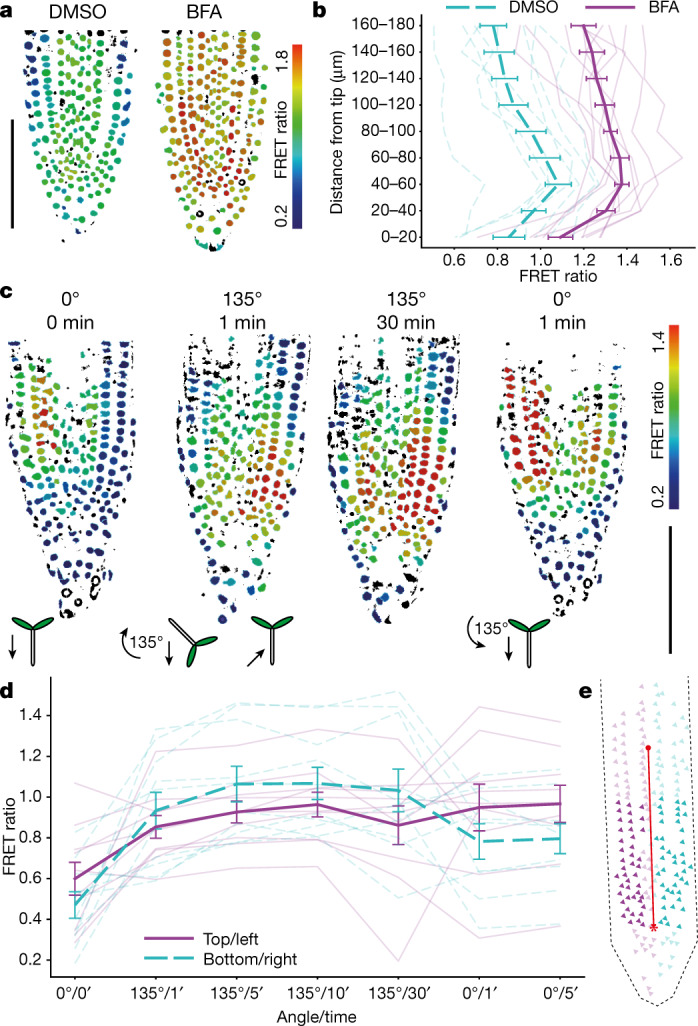
FRET ratio of auxin sensor in response to redistribution of endogenous auxin. **a**, **b**, Change in the nuclear FRET ratio (colour bar) within the root tip of individual seedlings treated with brefeldin A (BFA) or DMSO. **a**, Images. **b**, Quantitative analysis after treatment with BFA (10 μM for 10 h, magenta; *n* = 10) or DMSO (control, green; *n* = 9). The thick lines represent the mean (±s.e.m.), the thin lines each represent individual seedlings. **c**, **d**, The response of AuxSen to root gravitropism. **c**, Top, nuclear FRET ratio (colour bar) before and after turning (first and second images), and before and after turning back to the near-vertical position (third and fourth images). Bottom, Cartoons of the seedlings, with arrows indicating the direction of the gravity vector. The signal moves from the left side to the bottom and back to the left side; the colour scale indicates the relative FRET ratio. **d**, Quantified response of the sensor in individual roots. The thick lines represent the mean (±s.e.m.), the thin lines each represent individual roots (*n* = 10). Scale bars, 100 μm (**a**, **c**). **e**, Diagram of root tip. Nuclei within 100 μm above the quiescent centre (asterisk) and at least 10 μm from the midline (red line) were analysed (dark triangles). Green, right/bottom; magenta, left/top.

The spatial distribution of endogenous auxin in the seedling root has been inferred from the steady-state expression levels of auxin-response reporters such as *pDR5:GFP* or *p35S:DII-VENUS*, which display a pronounced maximum at the quiescent centre of the root meristem near the root tip^6^. Consistent with this, the FRET ratio of our auxin sensor steadily increased towards the root tip, although there was no prominent maximum (Fig. 4a, b). To assess the contribution of transport to the steady-state distribution of auxin, we incubated the seedlings in brefeldin A, which impairs auxin transport by inhibiting the polar recycling of the auxin efflux transporter PIN1^24^. FRET ratios were increased in the tip of treated roots compared to untreated controls (Fig. 4a, b). This increase presumably resulted from ongoing IAA synthesis in the root tip while efflux was impaired, which is consistent with mass spectrometry analysis of IAA biosynthesis in cell-sorted GFP-expressing lines^20^ and with the expression of auxin-biosynthesis genes in the root tip^25^. We conclude that AuxSen reports the perturbation of endogenous auxin distribution, which highlights its specificity for the detection of auxin.

The redirection of root growth in response to changes in orientation of the gravity vector is a prime example of rapid auxin signalling, which involves redistribution of endogenous auxin within the root tip^26^. Relocalization of the auxin efflux transporter PIN3 from the (former) basal to the (former) lateral plasma membrane has been detected 2 min after turning the root to a horizontal position^27^. However, to our knowledge, no concomitant change in auxin distribution has been reported. The earliest change that has been detected, using the auxin-responsive degradation reporter DII-VENUS, was a reduction in fluorescence in the new lower side 30 min after the onset of gravity stimulation^28^. We equilibrated seedling roots mounted in a near-vertical position for 1 h, then changed their orientation by 135° and monitored the nuclear FRET ratios over time (Fig. 4c, d). Within 1 min, there was a distinct increase in FRET ratio on the new lower side of the root tip, corresponding to more than 80% of the maximum value reached after 5 min. We then returned the roots to the near-vertical position after 30 min, and detected substantial recovery of the pre-stimulation distribution of FRET signals within 1 min (Fig. 4c, d). Gravistimulation therefore elicits a fast and reversible response of the auxin transport system in the root tip. In conclusion, this auxin sensor reports the rapid and reversible redistribution of endogenous auxin accumulation.

Our design approach has yielded a new sensor, AuxSen, for the pervasive plant signalling molecule auxin. Starting from a tryptophan sensor, we optimized the affinity and specificity for the binding of IAA, and improved the signal intensity through choice of the FRET pair and optimization of the linkers. Our results provide a proof-of-principle that this detection system can visualize the dynamic redistribution of auxin as well as subcellular pools of auxin, which cannot be achieved with the auxin reporters that are currently in use. An example of insights into auxin dynamics that have been made possible by the resolving power of AuxSen is the contrast between the efficient uptake and the slower, conditional efflux of auxin. Furthermore, AuxSen enables changes in auxin distribution to be distinguished from changes in auxin response, which is a prerequisite for investigating the complex regulatory network that underlies the biological effects of this major signalling molecule in plant growth and development.

## Methods

No statistical methods were used to predetermine sample size. The experiments were not randomized and the investigators were not blinded to allocation during experiments and outcome assessment.

### Cloning of in-vitro auxin sensors

The TrpR sensors Trp-CTY and Trp-CTYT were gifts from W. Frommer (Addgene plasmids 13533 and 13534). The fluorophores tested were also donated: Aquamarine by F. Merola (Addgene plasmid 42888), Clover and mRuby2 by K. Beam (Addgene plasmid 49089), mKO1 by K. Thorn (Addgene plasmid 44642) and mCherry by M. Bayer^29^. eGFP was amplified from pGIIK NLS:3xEGFP^30^. mNeonGreen, mWasabi and mTFP1 were purchased from Allele Biotechnology and Pharmaceuticals, mKate2 and TagRFP were from Evrogen.

For the initial screening, we first used the Trp-CTY sensor and mutated the residues T44 and S88 individually to all possible amino acids. To this end, we generated primers with 15–16 bp overlaps around the exchanged amino acid codons. We first mutated the amino acids sequence randomly with a degenerate primer and screened 96 clones. Variants not found were then generated by targeted mutagenesis. For example, to generate all T44 variants we first used the primers CCTGATGCTGnnnCCAGATGAGCGCG and CGCGCTCATCTGGnnnCAGCATCAGG, to generate the missing T44C variant we then used the primers CCTGATGCTGtgtCCAGATGAGCGCG and CGCGCTCATCTGGacaCAGCATCAGG. The most promising candidates were introduced by targeted mutagenesis into Trp-CTYT and TrpR without fluorescent proteins, which were analysed by isothermal titration calorimetry (ITC) and structural studies. Trp-CTYT with mutations T44L and S88Y was generated in two steps: we first used the primers CCTGATGCTGctaCCAGATGAGCGCG and CGCGCTCATCTGGtagCAGCATCAGG to generate Trp-CTYT with T44L and then GATTACGCGTGGATCTAACtac-CTGAAAGCCGCGCCC and GGGCGCGGCTTTCAGgtaGTTAGATCCACGCGTAATC to generate Trp-CTYL with the mutations T44L and S88Y. To produce the recombinant proteins, we cloned the TrpR domain into the pET21 expression vector and generated the variants by targeted mutagenesis. This procedure was repeated with the most promising variants after each round. The primer sequences are available upon request.

The final variant was then codon-optimized for *Arabidopsis* and synthesized by Thermo Fisher Scientific GENEART. To allow an easy exchange of the fluorophores we added restriction enzyme sites at the ends: BamH1 and XhoI around the first fluorophore and ApaI and HindIII around the second. All fluorescent proteins were tested in the fluorophore I–TrpR–fluorophore II–TrpR configuration. Having identified Aquamarine and mNeonGreen as the optimal pair, we introduced all final binding-pocket variants into the backbone by site-directed mutagenesis.

### Ligands used for screening and testing

IAA, TRP, IAN, indole-3-carboxaldehyde, indole, indole-3-acetyl alanine, indole-3-acetyl aspartic acid, indole-3-acetamide, indole-3-ethanol, l-kynurenine, 2-oxindole-3-acetic acid, phenylalanine, picloram, tryptamine, (NH_4_)_2_SO_4_, CaCl_2_, DTT, H_2_O_2_, NH_4_NO_3_, KNO_3_ and yucasin were purchased from Sigma-Aldrich; 4-hydroxyindole-3-carbaldehyde and 5-hydroxyindole-3-carboxylic acid from Santa Cruz Biotechnology; 1-naphthaleneacetic acid, KCl and DMSO from Carl-Roth; 2,4-dichlorophenoxyacetic acid (2,4-D) from Alfa Aesar; indole-3-acetyl glucose from TRC; indole-3-butyric acid from Serva; NaCl from Merck; NPA from Supelco; and BFA from Thermo Fisher Scientific.

### Mutagenesis

Trp-CTY variants were generated by site-directed mutagenesis with degenerate or specific oligonucleotides purchased from Sigma-Aldrich. Amplification was carried out using Pfu polymerase (Thermo Fisher Scientific).

More than one thousand oligonucleotides were used; the sequences and resulting vector maps are available upon request. Each variant was sequenced and screened in crude extract of sonicated bacteria for IAA binding; promising candidates were confirmed as purified proteins. The linkers were generated by site-directed mutagenesis, including linkers with 15–16 bp overlap and 3–9 degenerated nucleotides in the middle. To generate linkers shorter than the original ones, parts were deleted, whereas for longer linkers a fixed sequence was inserted into the middle to reduce the risk of generating stop codons by having too many degenerate nucleotides in the sequence.

### Protein expression and purification for screening

For protein expression, bacteria were grown in the dark on plates with LB-agar supplemented with ampicillin for 3 days at room temperature. To measure crude extracts, we resuspended the bacteria in 20 mM MOPS pH 7.2, sonicated the suspension with an MS 73 probe (Bandelin) and centrifuged the sample with a tabletop centrifuge (Eppendorf). Protein extraction was performed with His Spin trap columns according to the manufacturer’s instructions (GE Healthcare). We resuspended the bacteria of one Petri dish in 2 ml binding buffer and sonicated with a MS 73 probe. Buffer exchange was performed with illustra NAP-25 columns (GE Healthcare). All measurements were performed in 20 mM MOPS (Sigma-Aldrich) with an Infinite F200 plate reader (Tecan).

#### Ligands used for crystallization and ITC

IAA, IAN and TRP used for crystallization and ITC were dissolved in 50 mM Tris/300 mM NaCl pH 8 buffer containing 1% DMSO if necessary.

### Protein purification for crystallography and ITC

After subcloning to pET21b(+), wild-type TrpR and all variants were expressed in *E. coli* BL21(DE3) and purified with a NiIMAC column and a subsequent Superdex-S75 gel-filtration column. All purification steps and measurements were based on the above 50 mM Tris/300 mM NaCl pH 8 buffer.

### Crystallization, data collection and processing

Crystals of wild-type TrpR and variants with different ligands were obtained by standard vapour diffusion in sitting drop plates. The crystals were cryoprotected if needed and flash-cooled in liquid nitrogen. Data for single crystals were collected at the synchrotron beamline PXII (Swiss Light Source) at 100 K and 0.5 degree images were recorded on a Pilatus 6 M detector. Only variant TrpR(M42F/T44L/T81M/N87G/S88Y)–IAA was recorded at MX Beamlines BL14.1 at BESSY II (Helmholtz-Zentrum Berlin für Materialien und Energie). Data were indexed, integrated and scaled with the program XDS and converted with XDSCONV^31^. Molecular replacement was performed with Phenix using the coordinates of wild-type TrpR (PDB ID: 1WRP^32^ or 1TRO^33^) as search model. Model building was performed using the program Coot^34^, and refinement was performed using Phenix^35^. Details on crystallization conditions, data and refinement statistics for all structures are summarized in Extended Data Table 2b and Supplementary Table 1.

#### ITC

ITC was performed using a VP-ITC (MicroCal). The protein concentration was adjusted to 74 μM and 730 μM ligand solutions were prepared using the above buffer containing 1% DMSO. Measurements were performed at 20 °C with a stirring speed of 300 rpm, reference power 15 μcal s^−1^ and spacing of 300 s between injections. The data were analysed using the MicroCal program. Binding data were derived from sigmoidal fits based on a one-site binding model from two measurements for each variant. Heat-of-dilution baselines for the ligands alone were subtracted from the experimental data as previously described^36^. The pH dependence of IAA binding to the variant TrpR(M42F/T44L/T81M/N87G/S88Y) (AuxSen) was measured on a NanoITC LV device with a stir rate of 300 rpm, 15 injections with 2 μl and 300 s spacing between injections. The 170 μl cell was overfilled with 400 μl protein to ensure air-free filling. The protein was added at a concentration of 100 μM and the ligand IAA at 1 mM at 20 °C. For each pH the data were recorded in the respective buffer: (i) 50 mM Tris pH 8.5, 300 mM NaCl; (ii) 50 mM Tris pH 8.0, 300 mM NaCl; (iii) 50 mM Tris pH 7.5, 300 mM NaCl; (iv) 50 mM Tris pH 7.0, 300 mM NaCl; (v) 50 mM MES pH 6.5, 300 mM NaCl; (vi) 50 mM MES pH 6.0, 300 mM NaCl; (vii) 50 mM MES pH 5.5, 300 mM NaCl; (viii) 50 mM citrate buffer pH 5.0, 300 mM NaCl; and (ix) 50 mM citrate buffer pH 4.5, 300 mM NaCl.

### Test of different FRET pairs

We tested pairs of Aquamarine, mCerulean3, mTFP1 and mTurquoise2 with Clover, Ypet and mNeonGreen; Aquamarine was additionally tested with eGFP and mWasabi, and mTFP1 with TagRFP. Furthermore, we tested mNeonGreen, Clover and Ypet with TagRFP and mRuby2. mKO1 was tested with mCherry, mKate2, mNeonGreen and mWasabi. TagRFP was also tested with mTFP1, mWasabi, mKate2 and mCherry.

### Constructs for in-vivo AuxSen experiments

For protoplast expression, we cloned the final version of the sensor into pJIT60^22^ (pJIT60-2xp35S:NLS:AuxSen).

For expression in transgenic plants, we cloned the in-vitro optimized AuxSen in constructs for conditional two-component expression, using the pBay-bar vector (a gift from M. Bayer^37^). pRPS5a-mGAL4-VP16-GR-UAS_NLS was amplified from a pGII plasmid and inserted into pBay-bar digested with KpnI and BamHI with Gibson Assembly (In-Fusion Cloning, Takara Bio Europe SAS) according to the manufacturer´s instructions. In a second step, AuxSen and ocs terminator were inserted into *pBay-bar pRPS5a-mGAL4-VP16-GR_UAS_NLS* digested with BamHI. To obtain the individual spectra, we replaced *AuxSen* by *mNeonGreen* or *Aquamarine*. These constructs were used for transforming plants and as a template for the unmix matrix in Fiji. To generate the ER-localized auxin sensor SP:AuxSen:HDEL, we removed the NLS from the nuclear AuxSen construct and inserted the signal peptide of an *Arabidopsis* vacuolar basic chitinase and the HDEL ER retention sequence^38^ in frame with the coding sequence N-terminally and C-terminally, respectively.

### Flow cytometry of protoplasts transiently expressing AuxSen

Protoplasts were prepared from suspension cell cultures and transfected as previously described^39^, using 12-ml PP tubes and 12 μg of construct pJIT60-2xp35S:NLS:AuxSen per 120 μl of protoplasts (3.5 × 10^6^ per ml) per transfection. On the next day, transfected protoplasts were pooled, filtered through 100-μm nylon mesh and split into 200-μl aliquots. Each IAA stock (in DMSO) was added 1:100 with a timing offset to account for the 5-min measurement cycle, ensuring a 1-h treatment for each sample, performed in triplicate. Cytometric analysis was set up on a Beckman Coulter MoFlo XDP (100 μm CytoNozzle, 30.5/30.0 psi, PBS sheath) to excite mNeonGreen at 488 nm (70 mW, elliptical focus) and capture peak FL1 (534/30) and shoulder FL2 (585/29) emission; Aquamarine at 405 nm excitation (100 mW, spherical focus) and capture peak FL9 (465/30) and shoulder FL10 (529/28) emission. Data were collected and processed using Summit 5.5 (Beckman Coulter). The main light-scattering gate was determined by identifying the population expressing the greatest amount of reporter. The FRET response was the ratio mean of FL10/FL9, with the auxin response moving towards FL10, directly calculated in Summit 5.5. Representative plots were drawn with FCS Express v.6.06.0033 (deNovo).

### Plant material and growth conditions

Wild-type *Arabidopsis thaliana* (accession Col-0) plants were used for transformation. Plants were grown on soil at 24 °C, 65% relative humidity under long-day conditions (16-h illumination and 8-h dark period). Seeds were surface-sterilized, stratified for 2 days at 4 °C and grown on half-strength Murashige and Skoog agar plates containing 1% sucrose (0.5MS + S) (Serva). After 1 week plants were transferred to soil.

For imaging, 4-day-old seedlings were transferred to 0.5MS+S 25 μM DEX agar plates, and 16 h later to microscope slides on which they were incubated in 0.5MS+S + IAA or DMSO (control) at the specified concentrations and for the indicated periods of time. To preserve field-of-view and optimal buffer exchange we fixed the cover slip and root with double-sided adhesive tape (Tesa, type 05338, Beiersdorf). To exchange the buffer, we completely emptied the slide on a paper tissue and refilled from the side with the pipette. For BFA treatment, seedlings were transferred to 0.5MS+S 25 μM DEX plates containing either 10 μM BFA or 0.1% (v/v) DMSO (control), and mounted on microscope slides 10 h later.

### Imaging

The imaging of seedling roots was performed with an LSM780 confocal laser scanning microscope, running ZEN 2.3 black SP1 as acquisition software (Zeiss) and using a Zeiss LD C-Apochromat 40×/1,1 W Korr for all experiments except the gravitropism experiments, which were recorded with a Zeiss Plan-Apochromat 20×/0.8. Spectral imaging was performed using the QUASAR detection unit on the same system: Aquamarine was excited for FRET ratio measurement at 405 nm using 5 fluorescent channels (419–455 nm, 454–491 nm, 490–526 nm, 525–562 nm and 561–598 nm); subsequently, mNeonGreen was imaged for segmentation of the regions of interest with excitation at 488 nm and detection of 3 fluorescent channels (490–526 nm, 525–562 nm and 561–598 nm). Gravitropism imaging requiring control over the direction of gravity was performed with a custom-made horizontal imaging kit that can be equipped on most inverted microscope stands for wide-field and confocal imaging. The kit consists of two pieces: a holder for the objective with a mirror for changing the direction of the optical axis of the system from a vertical direction to a horizontal direction (components from Thorlabs). The second piece is a rotatable, vertical sample holder that can be mounted into a standard multiwell plate holder (components from Fischertechnik).

We used spectral FRET^40^ to be able to control for influences from sources of autofluorescence, which can be abundant in plant tissues^41^. Spectral FRET therefore also has the advantage that the method can be adapted by adjusting the number of acquisition channels if other sources of autofluorescence are present in different plant tissues.

All analyses were performed using a current version of Fiji^42^. First, signals generated were linearly unmixed^43^ using J. Walter’s spectral unmixing plugin (https://imagej.nih.gov/ij/plugins/spectral-unmixing.html). The unmixing matrix was generated with mNeonGreen, and Aquamarine as fluorophore controls and a wild-type Col-0 control for background autofluorescence. After this, the images were manually thresholded on all channels to remove unspecific signals and saturated areas, regions of interest (ROIs) for the cell nuclei were automatically generated based on the 488 nm/490–526 nm-channel data using an adaptive threshold plugin (by Q. Tseng, https://sites.google.com/site/qingzongtseng/adaptivethreshold) and the ‘Watershed’ and ‘Analyze Particles’ functions of ImageJ. We analysed all pixels of the image only for the ER. The FRET ratio was calculated by spectral unmixing of the channels using ‘Spectral Unmix’ version 1.3 (by J. Walter, https://imagej.nih.gov/ij/plugins/spectral-unmixing.html) and a precomputed unmixing matrix (see above) yielding the Aquamarine and mNeonGreen emission for division. Unmixed ROIs were colour-coded using the ‘ROI Color Coder’ plugin (BAR library, by T. Ferreira, http://imagejdocu.tudor.lu/doku.php?id=macro:roi_color_coder). In general, the colour scales were adapted for each experiment best reflecting the differences. Nuclei consisting of areas that were too small or those that had unrealistic high FRET ratios (owing to insufficient Aquamarine signal) were omitted (reflected as black in the colour coding).

### Reporting summary

Further information on research design is available in the Nature Research Reporting Summary linked to this paper.

## Online content

Any methods, additional references, Nature Research reporting summaries, source data, extended data, supplementary information, acknowledgements, peer review information; details of author contributions and competing interests; and statements of data and code availability are available at 10.1038/s41586-021-03425-2.

### Supplementary information

Supplementary Tables This file contains Supplementary Table 1 (Crystallographic data) and Supplementary Table 2 (Flow cytometry statistics).

Reporting Summary

## Figures and Tables

**Extended Data Table 1:**
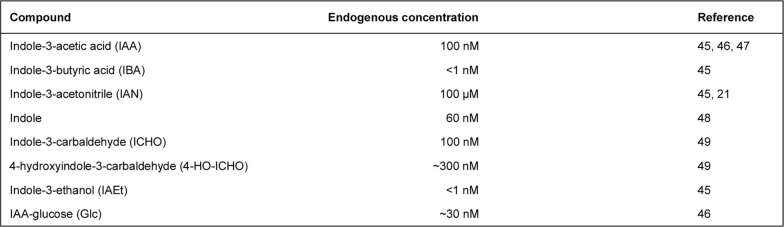
In vivo concentration of indole derivatives as reported in plant extracts. See refs. ^21,45–49^

**Extended Data Table 2:**
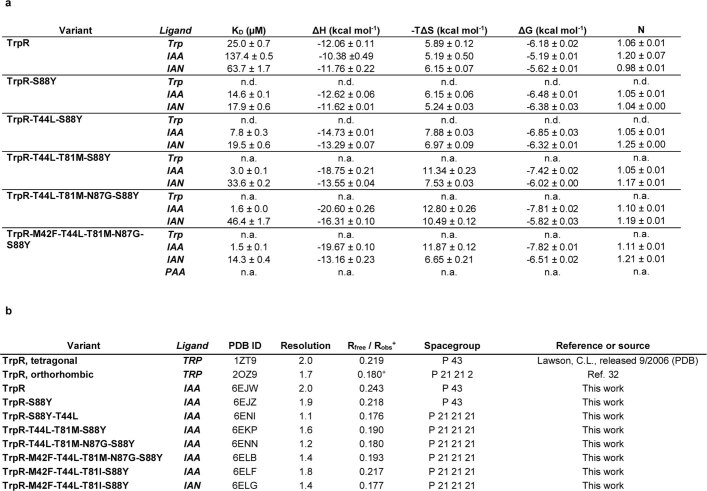
Dissociation constants and overview of crystallographic data for TrpR and selected variants

**a**, Dissociation constants of selected variants for TRP, IAA, IAN and PAA as determined by ITC. Errors are calculated from fits of two technical replicates. n.d. indicates that the data was measured but the fit did not converge owing to low affinity, whereas for later variants TRP measurements were omitted altogether (n.a.). The dissociation constants were determined via ITC, at 20 °C and pH 8.0. Thermodynamic values for enthalpy (Δ*H*), entropy (−*T*Δ*S*), and free energy (Δ*G*) are shown in kcal mol^−1^. Stoichiometry is given as *N*. Data are mean ± s.d., derived from 2 technical replicates.

**b**, Overview of crystallographic data for TrpR and its variants in complex with ligands as indicated.

## Data Availability

All processed data generated or analysed during this study are included in this Article and its Supplementary Information. Imaging (Herud_et_al_2021__figures_3_and_4_raw_data) and in vitro source data (Herud_et_al_2021__suppl_figures_S1_S3-S5_S7_and_table_S2a_raw_data) are available at Zenodo (10.5281/zenodo.4524537). The data for the FACS measurements in Fig. 3a, b and Extended Data Figs. 6, 7 can be found at https://flowrepository.org (FR-FCM-Z3FL). Coordinates and structure factors for all reported X-ray crystallography structures have been deposited in the PDB under accession codes 6EJW, 6EJZ, 6ENI, 6EKP, 6ENN, 6ELB, 6ELF and 6ELG. Materials (transgenic lines and plasmids) will be made available via the *Arabidopsis* Biological Resource Center (ABRC) in Columbus (OH, USA) and the Nottingham *Arabidopsis* Stock Centre (NASC), Nottingham (UK).

## Code availability

The ImageJ macros used to analyse data (Herud_et_al_2021__ImageJ_analysis_macros) are available from Zenodo (10.5281/zenodo.4524537).

## References

[1] Enders, T. A. & Strader, L. C. Auxin activity: Past, present, and future. *Am. J. Bot*. **102**, 180–196 (2015). 25667071 10.3732/ajb.1400285 PMC4854432

[2] Paque, S. & Weijers, D. Q&A: Auxin: the plant molecule that influences almost anything. *BMC Biol*. **14**, 67 (2016). 27510039 10.1186/s12915-016-0291-0 PMC4980777

[3] Ulmasov, T., Murfett, J., Hagen, G. & Guilfoyle, T. J. Aux/IAA proteins repress expression of reporter genes containing natural and highly active synthetic auxin response elements. *Plant Cell* **9**, 1963–1971 (1997). 9401121 10.1105/tpc.9.11.1963 PMC157050

[4] Friml, J. et al. Efflux-dependent auxin gradients establish the apical-basal axis of *Arabidopsis*. *Nature* **426**, 147–153 (2003). 14614497 10.1038/nature02085

[5] Weijers, D. et al. Auxin triggers transient local signaling for cell specification in *Arabidopsis* embryogenesis. *Dev. Cell* **10**, 265–270 (2006). 16459305 10.1016/j.devcel.2005.12.001

[6] Brunoud, G. et al. A novel sensor to map auxin response and distribution at high spatio-temporal resolution. *Nature* **482**, 103–106 (2012). 22246322 10.1038/nature10791

[7] Liao, C. Y. et al. Reporters for sensitive and quantitative measurement of auxin response. *Nat. Methods* **12**, 207–210 (2015). 25643149 10.1038/nmeth.3279 PMC4344836

[8] Marmorstein, R. Q., Joachimiak, A., Sprinzl, M. & Sigler, P. B. The structural basis for the interaction between l-tryptophan and the *Escherichia coli trp* aporepressor. *J. Biol. Chem*. **262**, 4922–4927 (1987). 3549712 10.1016/S0021-9258(18)61285-2

[9] Retzer, K., Butt, H., Korbei, B. & Luschnig, C. The far side of auxin signaling: fundamental cellular activities and their contribution to a defined growth response in plants. *Protoplasma* **251**, 731–746 (2014). 24221297 10.1007/s00709-013-0572-1 PMC4059964

[10] Leyser, O. Auxin signaling. *Plant Physiol*. **176**, 465–479 (2018). 28818861 10.1104/pp.17.00765 PMC5761761

[11] Ma, Q., Grones, P. & Robert, S. Auxin signaling: a big question to be addressed by small molecules. *J. Exp. Bot*. **69**, 313–328 (2018). 29237069 10.1093/jxb/erx375 PMC5853230

[12] Adamowski, M. & Friml, J. PIN-dependent auxin transport: action, regulation, and evolution. *Plant Cell* **27**, 20–32 (2015). 25604445 10.1105/tpc.114.134874 PMC4330589

[13] Bennett, T., Hines, G. & Leyser, O. Canalization: what the flux? *Trends Genet*. **30**, 41–48 (2014). 24296041 10.1016/j.tig.2013.11.001

[14] Naramoto, S. Polar transport in plants mediated by membrane transporters: focus on mechanisms of polar auxin transport. *Curr. Opin. Plant Biol*. **40**, 8–14 (2017). 28686910 10.1016/j.pbi.2017.06.012

[15] Pařízková, B., Pernisová, M. & Novák, O. What has been seen cannot be unseen—detecting auxin in vivo. *Int. J. Mol. Sci*. **18**, 2736 (2017). 29258197 10.3390/ijms18122736 PMC5751337

[16] Tsapakos, M. J., Haydock, P. V., Hermodson, M. & Somerville, R. L. Ligand-mediated conformational changes in Trp repressor protein of *Escherichia coli* probed through limited proteolysis and the use of specific antibodies. *J. Biol. Chem*. **260**, 16383–16394 (1985). 2415531 10.1016/S0021-9258(17)36248-8

[17] Zhang, R. G. et al. The crystal structure of *trp* aporepressor at 1.8 Å shows how binding tryptophan enhances DNA affinity. *Nature* **327**, 591–597 (1987). 3600756 10.1038/327591a0

[18] Kaper, T. et al. Nanosensor detection of an immunoregulatory tryptophan influx/kynurenine efflux cycle. *PLoS Biol*. **5**, e257 (2007). 17896864 10.1371/journal.pbio.0050257 PMC1988858

[19] Hamers, D., van Voorst Vader, L., Borst, J. W. & Goedhart, J. Development of FRET biosensors for mammalian and plant systems. *Protoplasma* **251**, 333–347 (2014). 24337770 10.1007/s00709-013-0590-z

[20] Petersson, S. V. et al. An auxin gradient and maximum in the *Arabidopsis* root apex shown by high-resolution cell-specific analysis of IAA distribution and synthesis. *Plant Cell* **21**, 1659–1668 (2009). 19491238 10.1105/tpc.109.066480 PMC2714926

[21] Katz, E. et al. The glucosinolate breakdown product indole-3-carbinol acts as an auxin antagonist in roots of *Arabidopsis thaliana*. *Plant J*. **82**, 547–555 (2015). 25758811 10.1111/tpj.12824

[22] Herud, O., Weijers, D., Lau, S. & Jürgens, G. Auxin responsiveness of the MONOPTEROS-BODENLOS module in primary root initiation critically depends on the nuclear import kinetics of the Aux/IAA inhibitor BODENLOS. *Plant J*. **85**, 269–277 (2016). 26714008 10.1111/tpj.13108

[23] Barbez, E. & Kleine-Vehn, J. *Divide Et Impera*—cellular auxin compartmentalization. *Curr. Opin. Plant Biol*. **16**, 78–84 (2013). 23200033 10.1016/j.pbi.2012.10.005

[24] Geldner, N. et al. The *Arabidopsis* GNOM ARF-GEF mediates endosomal recycling, auxin transport, and auxin-dependent plant growth. *Cell* **112**, 219–230 (2003). 12553910 10.1016/S0092-8674(03)00003-5

[25] Brumos, J. et al. Local auxin biosynthesis is a key regulator of plant development. *Dev. Cell* **47**, 306–318.e5 (2018). 30415657 10.1016/j.devcel.2018.09.022

[26] Su, S.-H., Gibbs, N. M., Jancewicz, A. L. & Masson, P. H. Molecular mechanisms of root gravitropism. *Curr. Biol*. **27**, R964–R972 (2017). 28898669 10.1016/j.cub.2017.07.015

[27] Friml, J., Wiśniewska, J., Benková, E., Mendgen, K. & Palme, K. Lateral relocation of auxin efflux regulator PIN3 mediates tropism in *Arabidopsis*. *Nature* **415**, 806–809 (2002). 11845211 10.1038/415806a

[28] Band, L. R. et al. Root gravitropism is regulated by a transient lateral auxin gradient controlled by a tipping-point mechanism. *Proc. Natl Acad. Sci. USA* **109**, 4668–4673 (2012). 22393022 10.1073/pnas.1201498109 PMC3311388

[29] Musielak, T. J., Slane, D., Liebig, C. & Bayer, M. A versatile optical clearing protocol for deep tissue imaging of fluorescent proteins in *Arabidopsis thaliana*. *PLoS ONE* **11**, e0161107 (2016). 27517463 10.1371/journal.pone.0161107 PMC4982668

[30] Takada, S. & Jürgens, G. Transcriptional regulation of epidermal cell fate in the *Arabidopsis* embryo. *Development* **134**, 1141–1150 (2007). 17301085 10.1242/dev.02803

[31] Kabsch, W. XDS. *Acta Crystallogr. D* **66**, 125–132 (2010). 20124692 10.1107/S0907444909047337 PMC2815665

[32] Lawson, C. L. et al. Flexibility of the DNA-binding domains of *trp* repressor. *Proteins* **3**, 18–31 (1988). 3375234 10.1002/prot.340030103

[33] Otwinowski, Z. et al. Crystal structure of *trp* repressor/operator complex at atomic resolution. *Nature* **335**, 321–329 (1988). 3419502 10.1038/335321a0

[34] Emsley, P. & Cowtan, K. Coot: model-building tools for molecular graphics. *Acta Crystallogr. D* **60**, 2126–2132 (2004). 15572765 10.1107/S0907444904019158

[35] Adams, P. D. et al. PHENIX: a comprehensive Python-based system for macromolecular structure solution. *Acta Crystallogr. D* **66**, 213–221 (2010). 20124702 10.1107/S0907444909052925 PMC2815670

[36] Machius, M. et al. Structural and biochemical basis for polyamine binding to the Tp0655 lipoprotein of *Treponema pallidum*: putative role for Tp0655 (TpPotD) as a polyamine receptor. *J. Mol. Biol*. **373**, 681–694 (2007). 17868688 10.1016/j.jmb.2007.08.018 PMC2094014

[37] Wang, K. et al. Independent parental contributions initiate zygote polarization in *Arabidopsis thaliana*. Preprint at 10.1101/2020.12.02.407874 (2020). 10.1016/j.cub.2021.08.033 34496220

[38] Haseloff, J., Siemering, K. R., Prasher, D. C. & Hodge, S. Removal of a cryptic intron and subcellular localization of green fluorescent protein are required to mark transgenic *Arabidopsis* plants brightly. *Proc. Natl Acad. Sci. USA* **94**, 2122–2127 (1997). 9122158 10.1073/pnas.94.6.2122 PMC20051

[39] Mehlhorn, D. G., Wallmeroth, N., Berendzen, K. W. & Grefen, C. 2in1 vectors improve in planta BiFC and FRET analyses. *Methods Mol. Biol*. **1691**, 139–158 (2018). 29043675 10.1007/978-1-4939-7389-7_11

[40] Piston, D. W. & Kremers, G. J. Fluorescent protein FRET: the good, the bad and the ugly. *Trends Biochem. Sci*. **32**, 407–414 (2007). 17764955 10.1016/j.tibs.2007.08.003

[41] Donaldson, L. Autofluorescence in plants. *Molecules* **25**, 2393 (2020). 32455605 10.3390/molecules25102393 PMC7288016

[42] Schindelin, J. et al. Fiji: an open-source platform for biological-image analysis. *Nat. Methods* **9**, 676–682 (2012). 22743772 10.1038/nmeth.2019 PMC3855844

[43] Neher, R. & Neher, E. Optimizing imaging parameters for the separation of multiple labels in a fluorescence image. *J. Microsc*. **213**, 46–62 (2004). 14678512 10.1111/j.1365-2818.2004.01262.x

[44] Lambert, T. J. FPbase: a community-editable fluorescent protein database. *Nat. Methods* **16**, 277–278 (2019). 30886412 10.1038/s41592-019-0352-8

[45] Novák, O. et al. Tissue-specific profiling of the *Arabidopsis thaliana* auxin metabolome. *Plant J*. **72**, 523–536 (2012). 22725617 10.1111/j.1365-313X.2012.05085.x

[46] Tam, Y. Y., Epstein, E. & Normanly, J. Characterization of auxin conjugates in *Arabidopsis*. Low steady-state levels of indole-3-acetyl-aspartate, indole-3-acetyl-glutamate, and indole-3-acetyl-glucose. *Plant Physiol*. **123**, 589–596 (2000). 10859188 10.1104/pp.123.2.589 PMC59026

[47] Mashiguchi, K. et al. The main auxin biosynthesis pathway in *Arabidopsis*. *Proc. Natl Acad. Sci. USA* **108**, 18512–18517 (2011). 22025724 10.1073/pnas.1108434108 PMC3215075

[48] Normanly, J., Cohen, J. D. & Fink, G. R. *Arabidopsis thaliana* auxotrophs reveal a tryptophan-independent biosynthetic pathway for indole-3-acetic acid. *Proc. Natl Acad. Sci. USA* **90**, 10355–10359 (1993). 8234297 10.1073/pnas.90.21.10355 PMC47773

[49] Böttcher, C. et al. The biosynthetic pathway of indole-3-carbaldehyde and indole-3-carboxylic acid derivatives in *Arabidopsis*. *Plant Physiol*. **165**, 841–853 (2014). 24728709 10.1104/pp.114.235630 PMC4044862

