# Supplementary material for: A biosensor for the direct visualization of auxin
Source: Nature. 2021 Apr 7;592(7856):768–72. doi: 10.1038/s41586-021-03425-2 (PMC8081663; doi:10.1038/s41586-021-03425-2)
Supplement: Supplementary file 1 — This file contains Supplementary Table 1 (Crystallographic data) and Supplementary Table 2 (Flow cytometry statistics). [file 41586_2021_3425_MOESM1_ESM.pdf]

nature portfolio

https://doi.org/10.1038/s41586-021-03425-2

Supplementary information

A biosensor for the direct visualization of auxin

In the format provided by the authors and unedited

SI Guide

A biosensor for direct visualisation of auxin

Ole Herud-Sikimić[1]*, Andre C. Stiel[1,2]*, Martina Kolb[1]*, Sooruban Shanmugaratnam[1,3],

Kenneth W. Berendzen[4], Christian Feldhaus[1], Birte Höcker[1,3]^, Gerd Jürgens[1]^

Suppl. Table S1.

Table S1a. Data collection and refinement statistics (molecular replacement) for TrpR-Wt with ligand IAA

Table S1b. Data collection and refinement statistics (molecular replacement) for TrpR-S88Y with ligand IAA

Table S1c. Data collection and refinement statistics (molecular replacement) for TrpR-T44L-S88Y with ligand IAA

Table S1d. Data collection and refinement statistics (molecular replacement) for TrpR-T44L-T81M-S88Y with ligand IAA

Table S1e. Data collection and refinement statistics (molecular replacement) for TrpR-T44L-T81M-N87G-S88Y with ligand IAA

Table S1f. Data collection and refinement statistics (molecular replacement) for TrpR-M42F-T44L-T81M-N87G-S88Y with ligand IAA

Table S1g. Data collection and refinement statistics (molecular replacement) for TrpR-M42F-T44L-T81I-S88Y with ligand IAA

Table S1h. Data collection and refinement statistics (molecular replacement) for TrpR-M42F-T44L-T81I-S88Y with ligand IAN

Suppl. Table S2.

Table S2. Flow cytometry statistics

Table S1a Data collection and refinement statistics (molecular replacement) for TrpR-Wt with ligand IAA

	TrpR-Wt IAA: 6EJW
**Data collection**	
Space group	P 4_3
Cell dimensions	
a, *b*, *c* (Å)	81.31 81.31 72.00
α, β, γ (°)	90 90 90
Resolution (Å)	44.93 - 1.99 (2.06 - 1.99) [a]
R_{sym} or R_{merge}	0.094 (1.962)
$I / \sigma I$	11.55 (0.91)
Completeness (%)	99.45 (96.48)
Redundancy	6.5 (6.0)
**Refinement**	
Resolution (Å)	44.93 - 1.99 (2.06 - 1.99)
No. reflections	32088 (3099)
R_{work} / R_{free}	0.195 (0.309) / 0.243 (0.364)
No. atoms	
Protein	3293
Ligand/ion	130
Water	130
B-factors	
Protein	50.2
Ligand/ion	46.4
Water	51.5
R.m.s. deviations	
Bond lengths (Å)	0.008
Bond angles (°)	0.90

[a] Numbers in brackets correspond to values in the highest resolution shell.

Table S1b Data collection and refinement statistics (molecular replacement) for TrpR-S88Y with ligand IAA

	TrpR-S88Y IAA: 6EJZ
**Data collection**	
Space group	P 4_3
Cell dimensions	
a, *b*, *c* (Å)	82.44 82.44 75.65
α, β, γ (°)	90 90 90
Resolution (Å)	41.22 - 1.90 (1.97 - 1.90) [a]
R_{sym} or R_{merge}	0.081 (1.816)
$I / \sigma I$	20.28 (1.47)
Completeness (%)	99.79 (98.67)
Redundancy	12.8 (12.6)
**Refinement**	
Resolution (Å)	41.22 - 1.90 (1.97 - 1.90)
No. reflections	40085 (3931)
R_{work} / R_{free}	0.184 (0.305) / 0.218 (0.337)
No. atoms	
Protein	3337
Ligand/ion	118
Water	220
B-factors	
Protein	43.8
Ligand/ion	49.4
Water	46.8
R.m.s. deviations	
Bond lengths (Å)	0.006
Bond angles (°)	0.69

[a] Numbers in brackets correspond to values in the highest resolution shell.

Table S1c Data collection and refinement statistics (molecular replacement) for TrpR-T44L-S88Y with ligand IAA

	TrpR-T44L-S88Y IAA: 6ENI
**Data collection**	
Space group	$P\ 2_1\ 2_1\ 2_1$
Cell dimensions	
a, *b*, *c* (Å)	54.78 63.41 64.77
α, β, γ (°)	90 90 90
Resolution (Å)	34.91 - 1.10 (1.14 - 1.10) [a]
R_{sym} or R_{merge}	0.052 (0.701)
$I / \sigma I$	26.67 (3.85)
Completeness (%)	94.44 (80.40)
Redundancy	12.7 (11.4)
**Refinement**	
Resolution (Å)	34.91 - 1.10 (1.14 - 1.10)
No. reflections	87117 (7337)
R_{work} / R_{free}	0.158 (0.263) / 0.176 (0.263)
No. atoms	
Protein	1805
Ligand/ion	30
Water	469
B-factors	
Protein	14.1
Ligand/ion	13.1
Water	23.7
R.m.s. deviations	
Bond lengths (Å)	0.010
Bond angles (°)	1.08

[a] Numbers in brackets correspond to values in the highest resolution shell.

Table S1d Data collection and refinement statistics (molecular replacement) for TrpR-T44L-T81M-S88Y with ligand IAA

	TrpR-T44L-T81M-S88Y IAA: 6EKP
**Data collection**	
Space group	$P\ 2_1\ 2_1\ 2_1$
Cell dimensions	
a, *b*, *c* (Å)	55.04 63.12 64.64
α, β, γ (°)	90 90 90
Resolution (Å)	41.48 - 1.46 (1.51 - 1.46) [a]
R_{sym} or R_{merge}	0.034 (0.770)
$I / \sigma I$	20.30 (1.97)
Completeness (%)	98.54 (94.76)
Redundancy	4.3 (4.2)
**Refinement**	
Resolution (Å)	41.48 - 1.46 (1.51 - 1.46)
No. reflections	39383 (3744)
R_{work} / R_{free}	0.172 (0.306) / 0.190 (0.332)
No. atoms	
Protein	1689
Ligand/ion	41
Water	250
B-factors	
Protein	35.0
Ligand/ion	30.8
Water	45.4
R.m.s. deviations	
Bond lengths (Å)	0.002
Bond angles (°)	0.50

[a] Numbers in brackets correspond to values in the highest resolution shell.

Table S1e Data collection and refinement statistics (molecular replacement) for TrpR-T44L-T81M-N87G-S88Y with ligand IAA

	TrpR-T44L-T81M-N87G-S88Y IAA: 6ENN
**Data collection**	
Space group	P 2_1 2_1 2_1
Cell dimensions	
a, *b*, *c* (Å)	54.58 63.19 64.90
α, β, γ (°)	90 90 90
Resolution (Å)	41.77 - 1.17 (1.21 - 1.17) [a]
R_{sym} or R_{merge}	0.052 (0.659)
$I / \sigma I$	16.16 (1.90)
Completeness (%)	98.04 (87.61)
Redundancy	5.8 (4.1)
**Refinement**	
Resolution (Å)	41.77 - 1.17 (1.21 - 1.17)
No. reflections	74760 (6616)
R_{work} / R_{free}	0.148 (0.225) / 0.180 (0.264)
No. atoms	
Protein	1837
Ligand/ion	26
Water	347
B-factors	
Protein	15.7
Ligand/ion	12.8
Water	30.9
R.m.s. deviations	
Bond lengths (Å)	0.010
Bond angles (°)	1.10

[a] Numbers in brackets correspond to values in the highest resolution shell.

Table S1f Data collection and refinement statistics (molecular replacement) for TrpR-M42F-T44L-T81M-N87G-S88Y with ligand IAA

	TrpR-M42F-T44L-T81M-N87G-S88Y IAA: 6ELB
**Data collection**	
Space group	P 2_1 2_1 2_1
Cell dimensions	
a, *b*, *c* (Å)	55.09 63.31 65.05
α, β, γ (°)	90 90 90
Resolution (Å)	45.37 - 1.44 (1.49 - 1.44) [a]
R_{sym} or R_{merge}	0.048 (1.086)
I / σI	19.27 (1.37)
Completeness (%)	98.25 (93.32)
Redundancy	7.2 (6.9)
**Refinement**	
Resolution (Å)	45.37 - 1.44 (1.49 - 1.44)
No. reflections	41354 (3869)
R_{work} / R_{free}	0.154 (0.227) / 0.193 (0.316)
No. atoms	
Protein	1771
Ligand/ion	26
Water	309
B-factors	
Protein	24.3
Ligand/ion	18.7
Water	36.4
R.m.s. deviations	
Bond lengths (Å)	0.006
Bond angles (°)	0.82

[a] Numbers in brackets correspond to values in the highest resolution shell.

Table S1g Data collection and refinement statistics (molecular replacement) for TrpR-M42F-T44L-T81I-S88Y with ligand IAA

	TrpR- M42F-T44L-T81I-S88Y IAA: 6ELF
**Data collection**	
Space group	P 2_1 2_1 2_1
Cell dimensions	
a, *b*, *c* (Å)	54.96 63.41 64.82
α, β, γ (°)	90 90 90
Resolution (Å)	31.70 - 1.83 (1.90 - 1.83) [a]
R_{sym} or R_{merge}	0.094 (1.985)
I / σI	18.08 (1.22)
Completeness (%)	99.50 (96.29)
Redundancy	12.5 (11.2)
**Refinement**	
Resolution (Å)	31.70 - 1.83 (1.90 - 1.83)
No. reflections	20416 (1945)
R_{work} / R_{free}	0.177 (0.409) / 0.217 (0.421)
No. atoms	
Protein	1701
Ligand/ion	26
Water	155
B-factors	
Protein	35.6
Ligand/ion	31.3
Water	42.4
R.m.s. deviations	
Bond lengths (Å)	0.013
Bond angles (°)	1.20

[a] Numbers in brackets correspond to values in the highest resolution shell.

Table S1h Data collection and refinement statistics (molecular replacement) for TrpR-M42F-T44L-T81I-S88Y with ligand IAN

	TrpR- M42F-T44L-T81I-S88Y IAN: 6ELG
**Data collection**	
Space group	P 2_1 2_1 2_1
Cell dimensions	
a, *b*, *c* (Å)	55.01 63.47 65.36
α, β, γ (°)	90 90 90
Resolution (Å)	42.09 - 1.38 (1.43 - 1.38)[a]
R_{sym} or R_{merge}	0.063 (0.772)
$I / \sigma I$	16.65 (1.83)
Completeness (%)	99.55 (96.20)
Redundancy	6.3 (5.8)
**Refinement**	
Resolution (Å)	42.09 - 1.38 (1.43 - 1.38)
No. reflections	47907 (4557)
R_{work} / R_{free}	0.143 (0.239) / 0.177 (0.295)
No. atoms	
Protein	1790
Ligand/ion	24
Water	387
B-factors	
Protein	16.8
Ligand/ion	12.8
Water	29.8
R.m.s. deviations	
Bond lengths (Å)	0.010
Bond angles (°)	0.99

[a] Numbers in brackets correspond to values in the highest resolution shell.

Table S2. Flow cytometry statistics

Sample description	Y Param	Gate	Mean Y	Median Y	Gate2	% Hist	Sample #	Sample	replicate	Dilution	Conc [1M]	FRET-ratio meanY	Conc [1M]	% Hist FRET-ratio	Conc [1M]	FRET-ratio meanY nz	Conc [1M]	FRET-ratio %
Sample_189_H2O	(FL10-Log_Height/FL9-Log_Height)	Total	0	0	FRET-RESPONSE	0	189	H2O	x	0	0	0	0	0	0	0,00	0	0,00
Sample_190_AQUA	(FL10-Log_Height/FL9-Log_Height)	Total	290,88	295,52	FRET-RESPONSE	66,67	190	AQUA	x	0	0	290,88	0	66,67	0	0,91	0	2,94
Sample_191_NEON	(FL10-Log_Height/FL9-Log_Height)	Total	394,34	338,39	FRET-RESPONSE	4,18	191	NEON	x	0	0	394,34	0	4,18	0	1,24	0	0,18
Sample_192_GFP	(FL10-Log_Height/FL9-Log_Height)	Total	987,72	666,08	FRET-RESPONSE	17,03	192	GFP	x	0	0	987,72	0	17,03	0	3,11	0	0,75
Sample_193_AUX_0_0	(FL10-Log_Height/FL9-Log_Height)	Total	312,95	309,17	FRET-RESPONSE	21,38	193	AUX	x	0	0	312,95	0	21,38	0	0,98	0	0,94
Sample_194_AUX_a_1	(FL10-Log_Height/FL9-Log_Height)	Total	749,86	636,68	FRET-RESPONSE	27,26	194	AUX	a	1	0,01	749,86	0,01	27,26	0,01	2,36	0,01	1,20
Sample_195_AUX_a_2	(FL10-Log_Height/FL9-Log_Height)	Total	670,34	608,57	FRET-RESPONSE	28,34	195	AUX	a	2	0,0025	670,34	0,0025	28,34	0,0025	2,11	0,0025	1,25
Sample_196_AUX_a_3	(FL10-Log_Height/FL9-Log_Height)	Total	597,79	556,03	FRET-RESPONSE	26,88	196	AUX	a	3	0,000625	597,79	0,000625	26,88	0,000625	1,88	0,000625	1,18
Sample_197_AUX_a_4	(FL10-Log_Height/FL9-Log_Height)	Total	452,96	424,08	FRET-RESPONSE	25,66	197	AUX	a	4	0,00015625	452,96	0,00015625	25,66	0,00015625	1,42	0,00015625	1,13
Sample_198_AUX_a_5	(FL10-Log_Height/FL9-Log_Height)	Total	377,88	370,36	FRET-RESPONSE	23,66	198	AUX	a	5	3,90625E-05	377,88	3,90625E-05	23,66	3,90625E-05	1,19	3,90625E-05	1,04
Sample_199_AUX_a_6	(FL10-Log_Height/FL9-Log_Height)	Total	340,12	338,39	FRET-RESPONSE	23,64	199	AUX	a	6	9,76563E-06	340,12	9,76563E-06	23,64	9,76563E-06	1,07	9,76563E-06	1,04
Sample_200_AUX_a_7	(FL10-Log_Height/FL9-Log_Height)	Total	330,7	323,45	FRET-RESPONSE	23,39	200	AUX	a	7	2,44141E-06	330,7	2,44141E-06	23,39	2,44141E-06	1,04	2,44141E-06	1,03
Sample_201_AUX_a_8	(FL10-Log_Height/FL9-Log_Height)	Total	325,35	323,45	FRET-RESPONSE	23,35	201	AUX	a	8	6,10352E-07	325,35	6,10352E-07	23,35	6,10352E-07	1,02	6,10352E-07	1,03
Sample_202_AUX_a_9	(FL10-Log_Height/FL9-Log_Height)	Total	320,86	323,45	FRET-RESPONSE	22,72	202	AUX	a	9	1,52588E-07	320,86	1,52588E-07	22,72	1,52588E-07	1,01	1,52588E-07	1,00
Sample_203_AUX_a_10	(FL10-Log_Height/FL9-Log_Height)	Total	326,59	323,45	FRET-RESPONSE	22,69	203	AUX	a	10	3,8147E-08	326,59	3,8147E-08	22,69	3,8147E-08	1,03	3,8147E-08	1,00
Sample_204_AUX_a_11	(FL10-Log_Height/FL9-Log_Height)	Total	321,15	323,45	FRET-RESPONSE	24,06	204	AUX	a	11	9,53674E-09	321,15	9,53674E-09	24,06	9,53674E-09	1,01	9,53674E-09	1,06
Sample_205_AUX_a_12	(FL10-Log_Height/FL9-Log_Height)	Total	324,87	323,45	FRET-RESPONSE	22,95	205	AUX	a	12	2,38419E-09	324,87	2,38419E-09	22,95	2,38419E-09	1,02	2,38419E-09	1,01
Sample_206_AUX_b_1	(FL10-Log_Height/FL9-Log_Height)	Total	737,1	636,68	FRET-RESPONSE	28,63	206	AUX	b	1	0,01	737,1	0,01	28,63	0,01	2,32	0,01	1,26
Sample_207_AUX_b_2	(FL10-Log_Height/FL9-Log_Height)	Total	673,65	608,57	FRET-RESPONSE	27,49	207	AUX	b	2	0,0025	673,65	0,0025	27,49	0,0025	2,12	0,0025	1,21
Sample_208_AUX_b_3	(FL10-Log_Height/FL9-Log_Height)	Total	593,06	556,03	FRET-RESPONSE	28,33	208	AUX	b	3	0,000625	593,06	0,000625	28,33	0,000625	1,86	0,000625	1,25
Sample_209_AUX_b_4	(FL10-Log_Height/FL9-Log_Height)	Total	461,27	443,67	FRET-RESPONSE	26,26	209	AUX	b	4	0,00015625	461,27	0,00015625	26,26	0,00015625	1,45	0,00015625	1,16
Sample_210_AUX_b_5	(FL10-Log_Height/FL9-Log_Height)	Total	376,13	370,36	FRET-RESPONSE	24,93	210	AUX	b	5	3,90625E-05	376,13	3,90625E-05	24,93	3,90625E-05	1,18	3,90625E-05	1,10
Sample_211_AUX_b_6	(FL10-Log_Height/FL9-Log_Height)	Total	343,42	338,39	FRET-RESPONSE	23,94	211	AUX	b	6	9,76563E-06	343,42	9,76563E-06	23,94	9,76563E-06	1,08	9,76563E-06	1,06
Sample_212_AUX_b_7	(FL10-Log_Height/FL9-Log_Height)	Total	327,81	323,45	FRET-RESPONSE	23,5	212	AUX	b	7	2,44141E-06	327,81	2,44141E-06	23,5	2,44141E-06	1,03	2,44141E-06	1,04
Sample_213_AUX_b_8	(FL10-Log_Height/FL9-Log_Height)	Total	320,02	323,45	FRET-RESPONSE	22,93	213	AUX	b	8	6,10352E-07	320,02	6,10352E-07	22,93	6,10352E-07	1,01	6,10352E-07	1,01
Sample_214_AUX_b_9	(FL10-Log_Height/FL9-Log_Height)	Total	320,66	323,45	FRET-RESPONSE	22,96	214	AUX	b	9	1,52588E-07	320,66	1,52588E-07	22,96	1,52588E-07	1,01	1,52588E-07	1,01
Sample_215_AUX_b_10	(FL10-Log_Height/FL9-Log_Height)	Total	322,21	323,45	FRET-RESPONSE	23,1	215	AUX	b	10	3,8147E-08	322,21	3,8147E-08	23,1	3,8147E-08	1,01	3,8147E-08	1,02
Sample_216_AUX_b_11	(FL10-Log_Height/FL9-Log_Height)	Total	321,47	323,45	FRET-RESPONSE	23,33	216	AUX	b	11	9,53674E-09	321,47	9,53674E-09	23,33	9,53674E-09	1,01	9,53674E-09	1,03
Sample_217_AUX_b_12	(FL10-Log_Height/FL9-Log_Height)	Total	323,82	323,45	FRET-RESPONSE	23,43	217	AUX	b	12	2,38419E-09	323,82	2,38419E-09	23,43	2,38419E-09	1,02	2,38419E-09	1,03
Sample_218_AUX_c_1	(FL10-Log_Height/FL9-Log_Height)	Total	731,46	636,68	FRET-RESPONSE	28,08	218	AUX	c	1	0,01	731,46	0,01	28,08	0,01	2,30	0,01	1,24
Sample_219_AUX_c_2	(FL10-Log_Height/FL9-Log_Height)	Total	669,51	608,57	FRET-RESPONSE	28,21	219	AUX	c	2	0,0025	669,51	0,0025	28,21	0,0025	2,10	0,0025	1,24
Sample_220_AUX_c_3	(FL10-Log_Height/FL9-Log_Height)	Total	599,75	556,03	FRET-RESPONSE	28,05	220	AUX	c	3	0,000625	599,75	0,000625	28,05	0,000625	1,89	0,000625	1,24
Sample_221_AUX_c_4	(FL10-Log_Height/FL9-Log_Height)	Total	473,14	443,67	FRET-RESPONSE	26,17	221	AUX	c	4	0,00015625	473,14	0,00015625	26,17	0,00015625	1,49	0,00015625	1,15
Sample_222_AUX_c_5	(FL10-Log_Height/FL9-Log_Height)	Total	382,84	370,36	FRET-RESPONSE	25,36	222	AUX	c	5	3,90625E-05	382,84	3,90625E-05	25,36	3,90625E-05	1,20	3,90625E-05	1,12
Sample_223_AUX_c_6	(FL10-Log_Height/FL9-Log_Height)	Total	341,03	338,39	FRET-RESPONSE	24,15	223	AUX	c	6	9,76563E-06	341,03	9,76563E-06	24,15	9,76563E-06	1,07	9,76563E-06	1,06
Sample_224_AUX_c_7	(FL10-Log_Height/FL9-Log_Height)	Total	326,56	323,45	FRET-RESPONSE	23,68	224	AUX	c	7	2,44141E-06	326,56	2,44141E-06	23,68	2,44141E-06	1,03	2,44141E-06	1,04
Sample_225_AUX_c_8	(FL10-Log_Height/FL9-Log_Height)	Total	318,71	309,17	FRET-RESPONSE	23,02	225	AUX	c	8	6,10352E-07	318,71	6,10352E-07	23,02	6,10352E-07	1,00	6,10352E-07	1,01
Sample_226_AUX_c_9	(FL10-Log_Height/FL9-Log_Height)	Total	319,9	323,45	FRET-RESPONSE	23,54	226	AUX	c	9	1,52588E-07	319,9	1,52588E-07	23,54	1,52588E-07	1,01	1,52588E-07	1,04
Sample_227_AUX_c_10	(FL10-Log_Height/FL9-Log_Height)	Total	322,59	323,45	FRET-RESPONSE	23,83	227	AUX	c	10	3,8147E-08	322,59	3,8147E-08	23,83	3,8147E-08	1,01	3,8147E-08	1,05
Sample_228_AUX_c_11	(FL10-Log_Height/FL9-Log_Height)	Total	318,1	309,17	FRET-RESPONSE	23,82	228	AUX	c	11	9,53674E-09	318,1	9,53674E-09	23,82	9,53674E-09	1,00	9,53674E-09	1,05
Sample_229_AUX_c_12	(FL10-Log_Height/FL9-Log_Height)	Total	319,87	309,17	FRET-RESPONSE	23,6	229	AUX	c	12	2,38419E-09	319,87	2,38419E-09	23,6	2,38419E-09	1,01	2,38419E-09	1,04
															min	318,10	min	22,69
