# Supplementary material for: A biosensor for the direct visualization of auxin
Source: Nature. 2021 Apr 7;592(7856):768–72. doi: 10.1038/s41586-021-03425-2 (PMC8081663; doi:10.1038/s41586-021-03425-2)
Supplement: Supplementary file 2 — Reporting Summary [file 41586_2021_3425_MOESM2_ESM.pdf]

nature research

Corresponding author(s): Gerd Juergens/Birte Hoecker

Last updated by author(s): 2021/02/24

Reporting Summary

Nature Research wishes to improve the reproducibility of the work that we publish. This form provides structure for consistency and transparency in reporting. For further information on Nature Research policies, see our Editorial Policies and the Editorial Policy Checklist.

Statistics

For all statistical analyses, confirm that the following items are present in the figure legend, table legend, main text, or Methods section.

n/a	Confirmed	
☐	☒	The exact sample size (*n*) for each experimental group/condition, given as a discrete number and unit of measurement
☐	☒	A statement on whether measurements were taken from distinct samples or whether the same sample was measured repeatedly
☒	☐	The statistical test(s) used AND whether they are one- or two-sided<br>*Only common tests should be described solely by name; describe more complex techniques in the Methods section.*
☒	☐	A description of all covariates tested
☒	☐	A description of any assumptions or corrections, such as tests of normality and adjustment for multiple comparisons
☐	☒	A full description of the statistical parameters including central tendency (e.g. means) or other basic estimates (e.g. regression coefficient) AND variation (e.g. standard deviation) or associated estimates of uncertainty (e.g. confidence intervals)
☒	☐	For null hypothesis testing, the test statistic (e.g. *F*, *t*, *r*) with confidence intervals, effect sizes, degrees of freedom and *P* value noted<br>*Give P values as exact values whenever suitable.*
☒	☐	For Bayesian analysis, information on the choice of priors and Markov chain Monte Carlo settings
☒	☐	For hierarchical and complex designs, identification of the appropriate level for tests and full reporting of outcomes
☒	☐	Estimates of effect sizes (e.g. Cohen's *d*, Pearson's *r*), indicating how they were calculated

Our web collection on statistics for biologists contains articles on many of the points above.

Software and code

Policy information about availability of computer code

Data collection	ZEN (Zeiss) / ZEN 2.3 black SP1<br>MicroCal VPViewer2000 1.04.0011<br>TA Instruments RunITC 3.4
Data analysis	ImageJ 1.53c / The ImageJ macros used to analyze data are available at “zenodo.org” as “10.5281/zenodo.4524537” ("Herud_et_al_2021__ImageJ_analysis_macros").<br>Spectral Unmixing Plugin Version 1.3 (Joachim Walter)<br>Adaptive Threshold Plugin (by Qingzong Tseng)<br>ROI Color Coder Plugin (BAR library 1.1.13, by Tiago Ferreira)<br>MicroCal LLC based on Origin 7.0<br>TA Instruments NanoAnalyze 3.7.0<br>XDS BUILT 20170215<br>XDSCONV BUILT 20170215<br>Phenix.Phaser 2.6.0<br>Phenix.refine 1.12_2829<br>Coot 0.8.9

For manuscripts utilizing custom algorithms or software that are central to the research but not yet described in published literature, software must be made available to editors and reviewers. We strongly encourage code deposition in a community repository (e.g. GitHub). See the Nature Research guidelines for submitting code & software for further information.

Data

Policy information about availability of data

All manuscripts must include a data availability statement. This statement should provide the following information, where applicable:

- Accession codes, unique identifiers, or web links for publicly available datasets
- A list of figures that have associated raw data
- A description of any restrictions on data availability

All processed data generated or analysed during this study are included in this published article and its supplementary information files. Imaging ("Herud_et_al_2021__figures_3_and_4_raw_data") and in vitro source data („Herud_et_al_2021__suppl_figures_S1_S3-S5_S7_and_table_S2_raw_data) are available at “zenodo.org” as “10.5281/zenodo.4524537”. The data for the FACS measurements in Fig. 3a and b and Extended data Figures S6 and S7 can be found at “flowrepository.org” as “FR-FCM-Z3FL”. Coordinates and structure factors for all reported X-ray crystallography have been deposited as 6EJW, 6EJZ, 6ENI, 6EKP, 6ENN, 6ELB, 6ELF, 6ELG at the “protein data bank” (pdb).

Field-specific reporting

Please select the one below that is the best fit for your research. If you are not sure, read the appropriate sections before making your selection.

☒ Life sciences ☐ Behavioural & social sciences ☐ Ecological, evolutionary & environmental sciences

For a reference copy of the document with all sections, see nature.com/documents/nr-reporting-summary-flat.pdf

Life sciences study design

All studies must disclose on these points even when the disclosure is negative.

Sample size	Sample sizes were chosen to yield clear results but were limited by the manual processing during the time course studies. Both treated and control samples comprised several seedlings (numbers indicated in the text) in each of which hundreds of nuclei were analysed in the root tip.
Data exclusions	No data were excluded from the analysis.
Replication	Experiments were repeated at least three times, involving different seedling populations on different days. All attempts at replication were successful.
Randomization	Individual 5 days old seedling populations of common ancestry were partitioned into two or three groups that were then treated in different ways.
Blinding	No blinding. Microscopic data acquisition was manual, interpretation was automatic involving the ImageJ macro.

Reporting for specific materials, systems and methods

We require information from authors about some types of materials, experimental systems and methods used in many studies. Here, indicate whether each material, system or method listed is relevant to your study. If you are not sure if a list item applies to your research, read the appropriate section before selecting a response.

Materials & experimental systems

n/a	Involved in the study	
☒	☐	Antibodies
☒	☐	Eukaryotic cell lines
☒	☐	Palaeontology and archaeology
☒	☐	Animals and other organisms
☒	☐	Human research participants
☒	☐	Clinical data
☒	☐	Dual use research of concern

Methods

n/a	Involved in the study	
☒	☐	ChIP-seq
☐	☒	Flow cytometry
☒	☐	MRI-based neuroimaging

Flow Cytometry

Plots

Confirm that:

☒ The axis labels state the marker and fluorochrome used (e.g. CD4-FITC).

☒ The axis scales are clearly visible. Include numbers along axes only for bottom left plot of group (a 'group' is an analysis of identical markers).

☒ All plots are contour plots with outliers or pseudocolor plots.

☒ A numerical value for number of cells or percentage (with statistics) is provided.

Methodology

Sample preparation	Arabidopsis cell-suspension-culture cells were protoplasted and transfected with FP reporter plasmids. The next day the cells were filtered (100µm) and treated with various IAA concentrations following the dilution scheme provided in Extended data Table S4.xlsx, 1 hour before in vivo flow cytometric analysis.
Instrument	XDP MoFlo Beckman Coulter
Software	Summit 5.5 (Beckman Coulter) was used for acquisition and analysis; FCS Express v6.06.0033 (deNovo) was used for plotting examples. The FRET-ratio calculation is carefully detailed in the Methods.
Cell population abundance	Only analysis was performed in the shown region (see Fig3, Fig.S6.).
Gating strategy	FSC/SSC gates of starting population marked in Ext data Fig S7a, boundaries between “positive” and “negative” cell populations indicated in Ext data Fig S7b-d

☒ Tick this box to confirm that a figure exemplifying the gating strategy is provided in the Supplementary Information.
